# Surfactant-Derived Nitrogen-Bridged MoS_2_/C Heterostructures for Robust Lithium-Ion Storage

**DOI:** 10.1007/s40820-026-02324-3

**Published:** 2026-08-23

**Authors:** Senchuan Huang, Kewei Pei, Yunyi Chen, Yangfei Cao, Jingwen Shangguan, Shiman He, Junxia Meng, Shanqing Zhang

**Affiliations:** 1 School of Chemical Engineering and Light Industry, Guangdong University of Technology, Guangzhou, 510006 People’s Republic of China; 2 School of Materials and Energy, Guangdong University of Technology, Guangzhou, 510006 People’s Republic of China; 3 School of Materials Science and Engineering, Guangdong Provincial Key Laboratory of Advanced Energy Storage Materials, South China University of Technology, Guangzhou, 510641 People’s Republic of China; 4 School of Physics and Electronics, Gannan Normal University, Ganzhou, 341000 People’s Republic of China; 5 School of Environment and Science, Griffith University, Gold Coast, QLD 4222 Australia

**Keywords:** Molybdenum disulfide, Porous materials, Lithium-ion storage, Surfactant transformation, Mo–N bridging

## Abstract

Structural stability, ionic transport, and electronic conductivity are the major challenges of layered transition-metal dichalcogenide/carbon nanocomposites (LTMD/C) for lithium-ion storage. Herein, to address these challenges, a quaternary ammonium surfactant-mediated strategy is proposed to simultaneously construct porous MoS_2_ architectures and in situ generate nitrogen-doped carbon (NC) layers chemically coupled to MoS_2_ via interfacial Mo–N bridges. The resultant N-bridged MoS_2_/C heterostructures (denoted as MoS_2_–N–C) exhibit excellent structural robustness, expanded interlayer spacing, and improved charge-transfer kinetics. As an anode in lithium-ion batteries (LIBs), the optimized MoS_2_–N–C_700_ electrode delivers remarkable cycling stability (~ 100% capacity retention after 800 cycles at 0.5 A g^−1^) and excellent rate capability. Moreover, lithium-ion supercapacitors (LISCs) based on the as-prepared MoS_2_–N–C composite achieve an ultrahigh power density of 3500 W kg^−1^, and excellent cycling stability over 10,000 cycles. The cycling performance in both LIBs and LISCs surpasses that of most previously reported MoS_2_/C nanocomposites and conventional carbon-based anodes. This surfactant-derived interfacial bridging structure design offers a general platform for developing robust LTMD/C heterostructured electrodes for energy storage systems.

**Graphical Abstract:**

**Supplementary Information:**

The online version contains supplementary material available at 10.1007/s40820-026-02324-3.

## Highlights

- A quaternary ammonium surfactant enables in situ formation of interfacial Mo–N bridges between MoS_2_ and carbon, resulting in robust and highly conductive heterointerfaces.
- Expanded MoS_2_ interlayer spacing and strong interfacial electronic coupling accelerate Li^+^ transport and electron conduction.
- Remarkable cycling stability with ~ 100% capacity retention over 800 cycles at 0.5 A g^−1^ for lithium-ion batteries, and an ultrahigh power density of 3500 W kg^−1^ and stable cycling over 10,000 cycles for lithium-ion supercapacitors.

**Supplementary Information:**

The online version contains supplementary material available at 10.1007/s40820-026-02324-3.

## Graphical abstract

**Figure:**
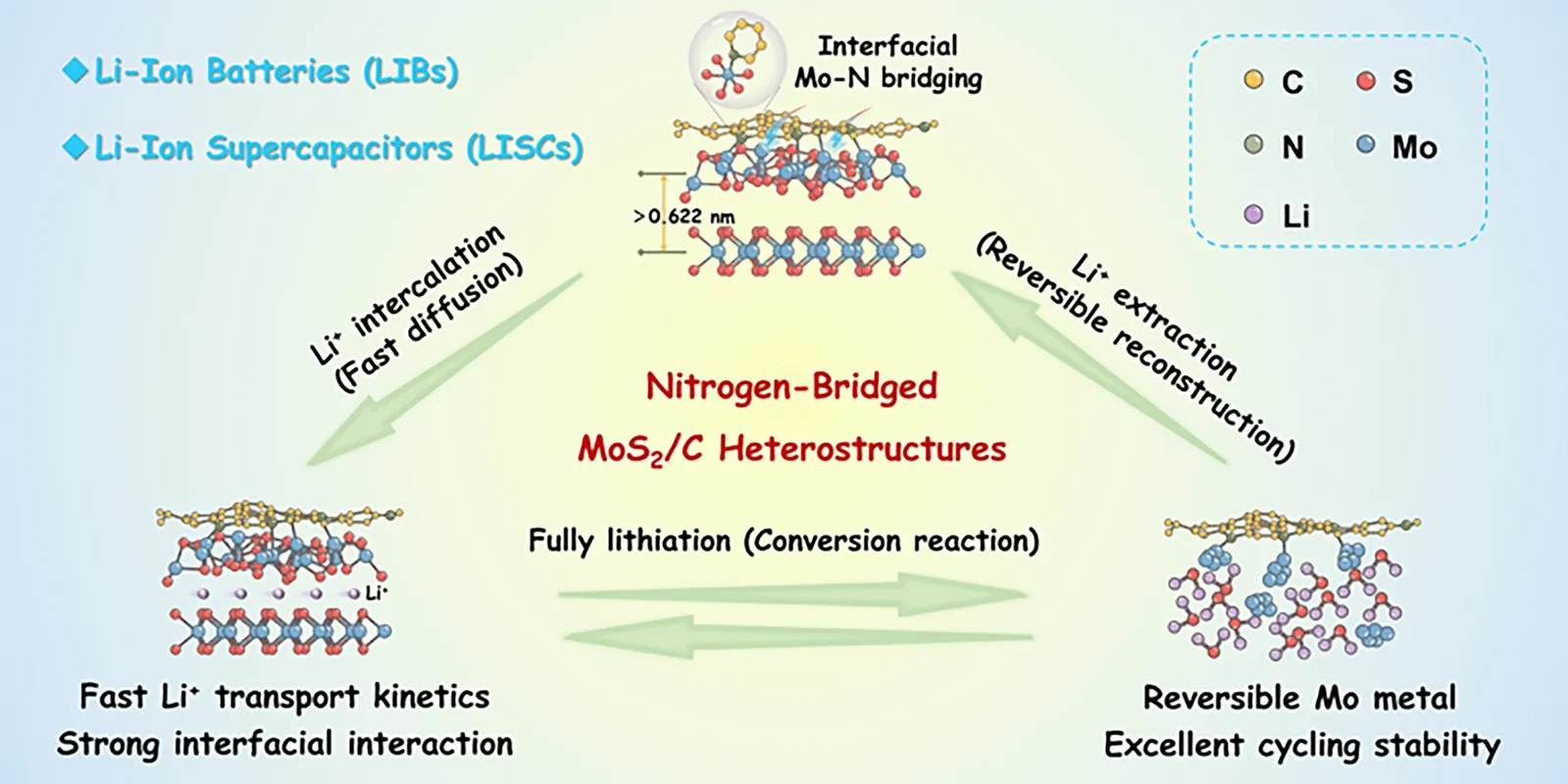

## Introduction

Large-scale energy storage systems that enable renewable integration and grid stability demand electrode materials with high capacity, long cycle life, and fast reaction kinetics [1–3]. Among various emerging candidates, layered transition-metal dichalcogenides (LTMDs), notably molybdenum disulfide (MoS_2_), have attracted considerable attention as anode materials because of their rich intercalation/conversion chemistry and high theoretical capacity of ~ 670 mAh g^−1^ for lithium-ion batteries (LIBs) [4]. However, the practical application of MoS_2_ is still limited by severe structural degradation during repeated lithiation/delithiation processes [5]. In addition, the intrinsically low electronic conductivity of semiconducting MoS_2_ restricts charge-transfer kinetics and rate performance [6].

**Scheme 1:**
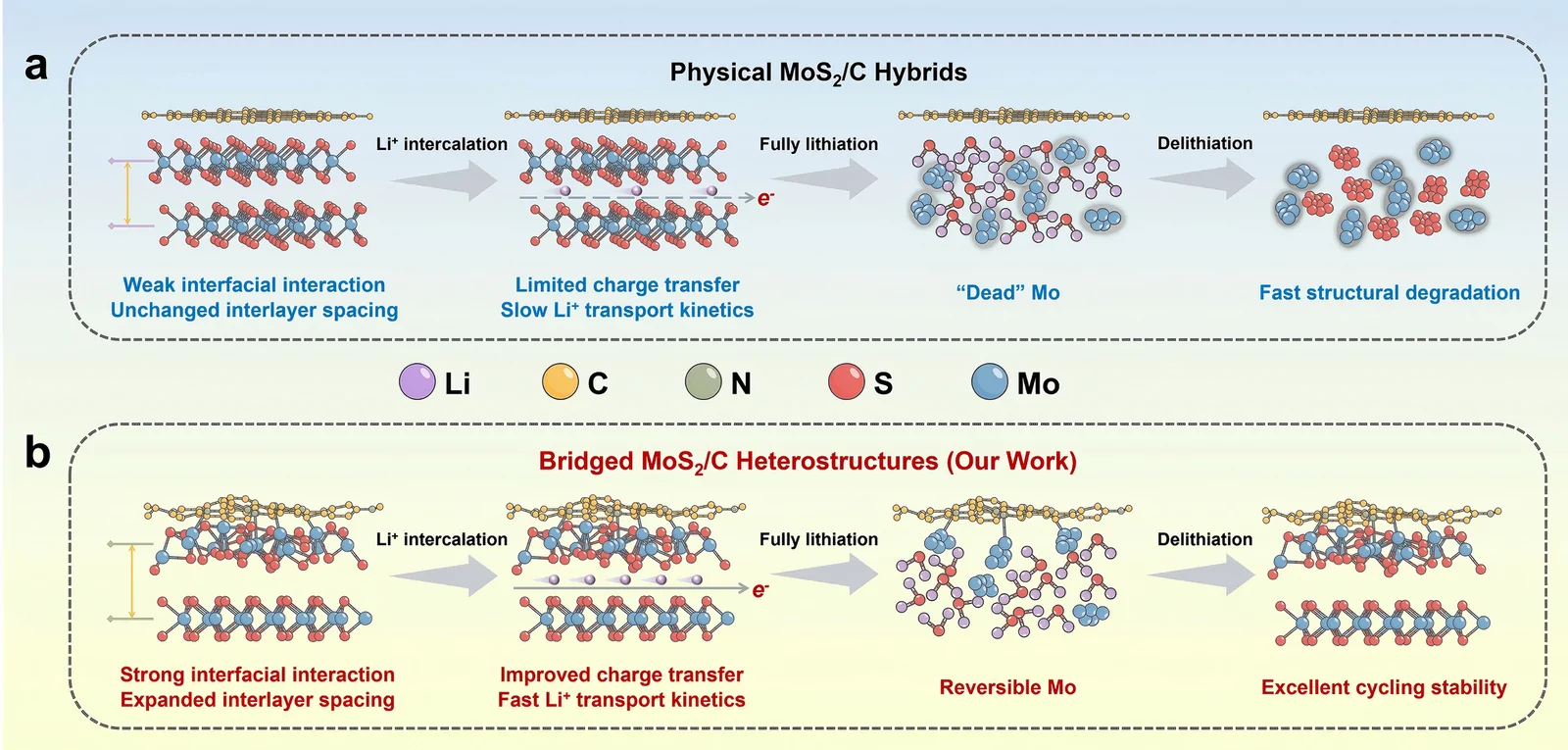
Schematics of **a** physical MoS_2_/C hybrids and **b** the proposed nitrogen-bridged MoS_2_/C heterostructures for electrochemical Li^+^ storage and their structure evolution during cycling

To mitigate these problems, extensive efforts have focused on morphology engineering [7], heteroatom doping [8, 9], and carbon hybridization [10, 11]. Compositing MoS_2_ with conductive carbon materials effectively improves electron transport and buffers volume changes, while further pore engineering and few-layer architectures promote Li^+^ accessibility [12, 13]. Nevertheless, most reported nanocomposites rely on weak physical contacts or simple surface coatings, which often leads to interfacial detachment, interlayer collapse, and progressive degradation during long-term cycling [14, 15]. As a result, in physical MoS_2_ and carbon hybrids (MoS_2_/C), MoS_2_ often undergoes slow Li^+^ transport and incomplete conversion reactions during lithiation. The formation of poorly conductive Li_2_S and electrochemically inactive (“dead”) Mo further impedes the reverse conversion process during delithiation, thereby causing severe structural degradation and rapid capacity decay during cycling (Scheme 1a). Moreover, multilayer MoS_2_ with MoS_2_/MoS_2_ interfaces is prone to pulverization after repeated cycling, which further deteriorates electrochemical reversibility.

Interfacial engineering that creates strong chemical bonds, such as Mo–N or Mo–C, between MoS_2_ and carbon offers a promising route to reconcile electronic conductivity, structural integrity, and reversible electrochemistry. Recent approaches, including heteroatom-doped carbons [16, 17], single-atom bridges [18, 19], and engineered 2D superlattices [20, 21], have demonstrated that interfacial chemistry and stacking configurations critically govern reaction kinetics and cycling durability. However, these approaches often require additional reagents and complex synthetic routes, raising concerns regarding cost and scalability [22]. Moreover, insufficient interlayer spacing caused by weak interfacial interactions continues to hinder Li⁺ diffusion kinetics in many existing systems [23, 24].

Herein, we introduce a quaternary ammonium surfactant-mediated strategy to construct porous MoS_2_/C heterostructures with interfacial Mo–N bridges, thereby improving the conversion reversibility of MoS_2_ during lithiation/delithiation. The Mo–N bridges serve not only as efficient electron-transfer pathways but also as robust structural anchors that preserve electrode integrity during cycling. The resulting N-bridged MoS_2_ and carbon heterostructure (denoted as MoS_2_–N–C) provides strong interfacial interaction and expanded interlayer spacing, thereby accelerating electron transfer and Li⁺ diffusion and converting into reversible Mo species for excellent cycling stability (Scheme 1b). Remarkably, the optimized MoS_2_–N–C_700_ heterostructure achieves ~ 100% capacity retention after 800 cycles at 0.5 A g^−1^ for LIBs. Moreover, when paired with activated carbon (AC) in lithium-ion supercapacitors (LISCs), it exhibits high power density and stable cycling for over 10,000 cycles at 1 A g^−1^, underscoring its strong potential as a next-generation high-performance anode material. This work demonstrates a scalable molecular-level strategy to construct chemically coupled LTMD/C heterostructures toward electrochemical energy storage systems.

## Experimental Section

### Materials

Cyclohexane (C_6_H_12_) and tetraethyl orthosilicate ((C_2_H_5_O)_4_Si, TEOS) were obtained from Aladdin. Cetyltrimethylammonium bromide (C_19_H_42_BrN, CTAB) was bought from Sinopharm Chemical Reagent Co., Ltd. Triethanolamine (C_6_H_15_NO_3_, TEA) and aniline were received from Sigma-Aldrich. Ammonium molybdate tetrahydrate ((NH_4_)_6_Mo_7_O_24_·4H_2_O, AMT), thiourea (CH_4_N_2_S), ammonium metatungstate ((NH_4_)_6_H_2_W_12_O_10_·*x*H_2_O), sodium hydroxide (NaOH), ammonium persulfate (APS), and commercial molybdenum disulfide (MoS_2_) were purchased from Shanghai Macklin Biochemical Co., Ltd. All chemical reagents were used as received without further purification. Deionized water was used throughout the experiments.

### Material Preparation

#### Preparation of CTAB-Decorated Mesoporous Silica (mSiO_2_@CTAB)

The synthesis of mSiO_2_@CTAB was carried out in an oil–water biphasic stratification reaction system, in which TEOS was used as a silica source for self-assembly at the oil–water interface, and CTAB served as a pore-forming agent in the aqueous phase. Specifically, 6 g of CTAB and 0.75 mL of TEA solution (0.3 g mL^−1^) were dissolved in 60 mL of deionized water and stirred at 300 r min^−1^ for 30 min at 60 °C. Afterward, 16 mL of cyclohexane and 4 mL of TEOS were added to the solution and stirred for 12h. The products were collected by centrifugation and washed three times with ethanol to remove unreacted species, while preserving CTAB in the nanopores of mesoporous silica (mSiO_2_).

#### Preparation of Nitrogen-Doped Carbon Bridged MoS_2_ Heterostructures (MoS_2_–N–C)

Specifically, 0.3 g of mSiO_2_@CTAB and 4.1 g of AMT were dispersed in 20 mL of deionized water by ultrasonication until a uniform suspension was obtained. The mixture was then filtered under vacuum through a Büchner funnel three times. The collected product was dried overnight at 80 °C to yield mSiO_2_@CTAB-MoO_*x*_ hybrid precursor. Notably, the amount of AMT was calculated according to Eq. 1, under the assumption that the pore volume of mSiO_2_@CTAB was completely filled with MoO_3_, and an additional 50% excess was introduced to ensure sufficient loading:

1 $$m_{{{\mathrm{AMT}}}} = \frac{{V_{{{\mathrm{pore}}}} \times m_{{{\mathrm{mSiO}}_{{2}} {\mathrm{@CTAB}}}} \times \rho_{{{\mathrm{MoO}}_{{3}} }} \times {\mathrm{MW}}_{{{\mathrm{AMT}}}} }}{{7 \times {\mathrm{MW}}_{{{\mathrm{MoO}}_{{3}} }} }} \times \left( {1 + 50\% } \right)$$

where ${V}_{\mathrm{p}\mathrm{o}\mathrm{r}\mathrm{e}}$ is the pore volume of mSiO_2_@CTAB, ${m}_{{\mathrm{m}\mathrm{S}\mathrm{i}\mathrm{O}}_{2}@\mathrm{C}\mathrm{T}\mathrm{A}\mathrm{B}}$ is the amount of mSiO_2_@CTAB, ${\rho}_{{\mathrm{M}\mathrm{o}\mathrm{O}}_{3}}$ is the density of MoO_3_, ${\mathrm{M}\mathrm{W}}_{{\mathrm{M}\mathrm{o}\mathrm{O}}_{3}}$ is the molecular weight of MoO_3_, and ${\mathrm{M}\mathrm{W}}_{\mathrm{A}\mathrm{M}\mathrm{T}}$ is the molecular weight of AMT.

In the subsequent step, two separate alumina boats containing 1.2 g of thiourea and 0.6 g of mSiO_2_@CTAB-MoO_*x*_ hybrid precursor were placed at the upstream and downstream ends of a tube furnace, respectively, under an argon flow rate of 40 mL min^−1^. To minimize thiourea volatilization during heating, the boat containing thiourea was covered with an alumina lid. The samples were annealed at 600, 700, and 800 °C for 2h under an argon atmosphere to obtain the mSiO_2_@MoS_2_–N–C composites. After natural cooling, the mSiO_2_ template in the composites was removed by etching in 3 M NaOH aqueous solution. The as-synthesized materials were collected by centrifugation, thoroughly washed with deionized water, and dried overnight at 80 °C, yielding the final products denoted as MoS_2_–N–C_600_, MoS_2_–N–C_700_, and MoS_2_–N–C_800_, respectively.

Furthermore, nitrogen-doped carbon bridged WS_2_ heterostructures (WS_2_–N–C_700_) were obtained under identical conditions to MoS_2_–N–C_700_ by replacing 4.1 g of AMT with 3.5 g of ammonium metatungstate.

#### Preparation of Physical Hybrid of Nitrogen-Doped Carbon and MoS_2_ (MoS_2_/NC)

Nitrogen-doped carbon was derived from polyaniline. In detail, 1.6 mL of aniline was dispersed in 40 mL of ethanol and 10 mL of water, while 4.0 g of APS was dissolved in 10 mL of water. The APS solution was then added to the aniline solution. The mixture was kept stirring for 6h to finish the polymerization process. After centrifugation with water and ethanol alternatively, polyaniline was obtained and dried overnight at 80 °C for use. The product was pyrolyzed at 700 °C for 2h under Ar to yield nitrogen-doped carbon (NC). The ramping rate was 10 °C min^−1^. Finally, MoS_2_/NC was prepared by uniformly mixing commercial MoS_2_ with nitrogen-doped carbon at a weight ratio of 3:1 according to the TG analysis of MoS_2_–N–C_700_.

### Characterization

The morphology and microstructure were recorded through field emission scanning electron microscope (FESEM, Hitachi SU8220) and transmission electron microscope (TEM, FEI, Thermo Talos F200S). The crystal structures were analyzed by X-ray diffraction (XRD, Rigaku Ultima IV) equipped with Cu *K*α radiation (*λ* = 1.5418 Å). Raman spectra were collected on a HORIBA Jobin Yvon LabRAM HR Evolution spectrometer with a laser wavelength of 532 nm. Electron paramagnetic resonance (EPR) spectra were examined using Bruker EMXplus-10/12. Thermogravimetric (TG) analysis was conducted on a NETZSCH/STA449F5 instrument under an air atmosphere from 30 to 700 °C at a heating rate of 10 °C min^−1^. During TG measurements, the carbon component was completely oxidized into CO_2_, while MoS_2_ was converted into MoO_3_. Therefore, the final residual mass corresponds to MoO_3_. Based on the stoichiometric relationship between MoS_2_ and MoO_3_, the MoS_2_ content in the composite was calculated from the residual mass according to their molecular weights, and the carbon content was subsequently determined from the overall mass loss. The Brunauer–Emmett–Teller (BET) surface area was analyzed by nitrogen adsorption/desorption in an advanced specific surface area and micropore analyzer (BSD-660 M, Beishide Instrument). The pore size distribution plots were recorded from the adsorption branch of the isotherms based on the Barrett–Joyner–Halenda (BJH) model. X-ray photoelectron spectroscopy (XPS) measurement was performed on an ESCALAB 250 X-ray photoelectron spectrometer with an Al *K*α radiation source, with all binding energies referenced to the C 1*s* peak at 284.8 eV. X-ray absorption near-edge structure (XANES) spectra were collected using a laboratory-based XAFS instrument (easyXAFS300+ , easyXAFS LLC) equipped with a Rowland-circle geometry. An Ag-anode X-ray tube operated at 40 kV and 25 mA was employed as the excitation source, together with a Si (12 12 0) spherically bent crystal analyzer and a silicon drift detector (KETEK). The extended X-ray absorption fine structure (EXAFS) spectrum was extracted from the XANES data using Athena software.

### Electrochemical Measurements

The as-prepared electrode materials were mixed with Super P carbon as a conductive agent and polyvinylidene fluoride (PVDF) as a binder in N-methyl-2-pyrrolidone (NMP) at a mass ratio of 7:2:1 to form a uniform slurry. The slurry was coated onto copper foil substrates and dried under vacuum at 80 °C for 12 h. Circular disks with a diameter of 10 mm were then punched out as working electrodes. CR2032-type coin cells were assembled in an argon-filled glove box (oxygen and water are less than 0.01 ppm), employing Celgard 2500 polypropylene film (obtained from Canrd Technology Co., Ltd.) as the separator and 1 M LiPF_6_ dissolved in a mixed solvent of ethylene carbonate (EC), dimethyl carbonate (DMC), and ethyl methyl carbonate (EMC) (1:1:1 by volume) as the electrolyte. The mass loading of anode materials was 1.1–1.3 mg cm^−2^ unless otherwise stated.

To investigate the electrochemical properties of the electrode materials, galvanostatic charge/discharge (GCD) tests were performed at room temperature using a Neware battery testing system (MIHW-200-160CH-B) in the voltage window of 0.01–3.0 V (*vs.* Li/Li^+^). Cyclic voltammetry (CV) tests were carried out on a Princeton VersaSTAT 3F electrochemical workstation, employing scan rates from 0.1 to 1.0 mV s^−1^ to evaluate redox characteristics and reaction kinetics. Additionally, electrochemical impedance spectroscopy (EIS) measurements were conducted using a DH7000D electrochemical workstation (Donghua Analytical Instruments Co., Ltd.) with an amplified voltage of 5 mV, over a frequency range from 0.01 Hz to 100 kHz.

After activation at 0.1 A g^−1^ for 5 cycles, the galvanostatic intermittent titration technique (GITT) was employed to measure the Li^+^ diffusion coefficients (${D}_{{\mathrm{L}\mathrm{i}}^{+}}$). The procedure involved applying a series of pulse currents at 0.1 A g^−1^ for 10 min followed by relaxation intervals of 60 min. The ${D}_{{\mathrm{L}\mathrm{i}}^{+}}$ was determined by Eq. 2:

2 $$D_{{{\mathrm{Li}}^{ + } }} = \frac{4}{\pi \tau }\left( {\frac{{n_{{\mathrm{m}}} V_{{\mathrm{M}}} }}{S}} \right)^{2} \left( {\frac{{\Delta E_{s} }}{{\Delta E_{\tau } }}} \right)^{2} ;\left( {\tau \ll L^{2} /D_{{{\mathrm{Li}}^{ + } }} } \right)$$

where *τ* represents the constant current pulse time, and *n*_m_ and *V*_m_ are the molar number and molar volume of active material, respectively. *S* is the contact area of the electrode–electrolyte interface. ∆*E*_*s*_ and ∆*E*_τ_ represent the *iR* drop and the potential difference in a constant current pulse during the cycling process, respectively.

The relationship between current response (*i*) and scan rate (*v*) at the redox peaks in the CV curves was determined using Eq. 3:

3 $$i = av^{b}$$

where *a* and *b* are both constants. The value of *b*, which is equal to the slope of linear fit of log(*v*) versus log(*i*), indicates the type of process: A value of 0.5 suggests a diffusion-controlled (battery-like) process, whereas 1.0 indicates a pseudocapacitive (capacitor-like) behavior. The specific contributions from pseudocapacitive (*k*_1_*v*) and diffusion-controlled (*k*_2_*v*^1/2^) current responses can be quantitatively obtained by Eqs. 4 and 5:

4 $$i = k_{{1}} v + k_{{2}} v^{{{1}/{2}}}$$

or

5 $$i/v^{{{1}/{2}}} = k_{{1}} v^{{{1}/{2}}} + k_{{2}}$$

where *k*_1_ is equal to the slope of linear fit of *v*^1/2^ versus *i* / *v*^1/2^.

Device-level lithium-ion supercapacitors (LISCs) were assembled with activated carbon (AC, KURARAY YP-80F) as the cathode and the as-prepared MoS_2_–N–C_700_ as the anode. Prior to assembly, the anode material was prelithiated at 0.1 A g^−1^ for 5 GCD cycles. The LISCs were assembled following the same protocol as described for LIBs. The mass loading ratio of cathode to anode materials was optimized to 3:1 according to the specific capacities of the two electrodes to achieve charge balance. Such optimization ensures efficient utilization of both electrodes while effectively suppressing lithium plating on the anode side. The operating voltage window was set to 0.5–4.0 V based on the electrochemically stable potential ranges of both electrodes determined from half-cell measurements. The upper cutoff voltage was limited to 4.0 V to avoid parasitic oxidation reactions at higher potentials, which could otherwise compromise long-term cycling stability. Meanwhile, the lower cutoff voltage of 0.5 V confines the electrochemical reaction primarily within the intercalation regime, thereby mitigating excessive conversion reactions, preserving the structural integrity of MoS_2_, and enhancing cycling durability.

The specific capacitance (*C*) of the LISCs was calculated from the GCD curves using Eq. 6:

6 $$C = \frac{I \cdot \Delta t}{{m \cdot \Delta V}}$$

where *I* is the constant current applied during the charge/discharge process, Δ*t* represents the charge/discharge time, *m* is the mass of the cathode material, and Δ*V* denotes the operating voltage window of LISCs, ranging from 0.5 to 4.0 V.

The energy density (*E*) and power density (*P*) were calculated using Eqs. 7 and 8:

7 $$E = \frac{1}{2}C\Delta V^{2}$$

8 $$P = \frac{E}{\Delta t}$$

where ΔV is the voltage window, and Δt represents the charge/discharge time.

## Results and Discussion

### Surfactant-Mediated Synthesis and Structural Characterization

**Fig. 1:**
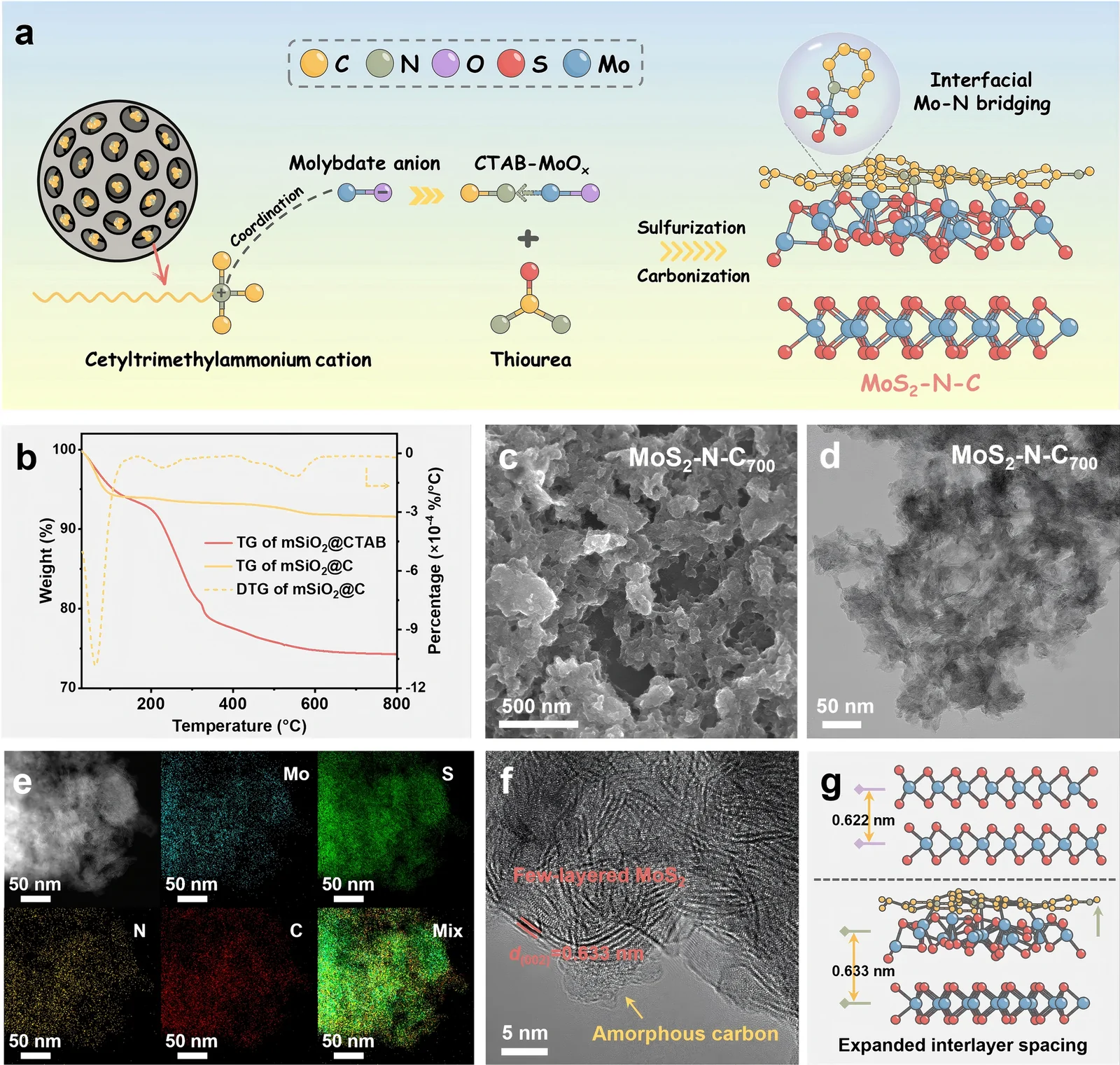
Synthesis and structural characterization of the MoS_2_–N–C heterostructures. **a** Schematic of the surfactant-mediated synthesis and formation of Mo–N-bridged carbon layers. **b** TG curves of mSiO_2_@CTAB under Ar and mSiO_2_@C in air, along with the DTG curve of mSiO_2_@C. **c** FESEM image, **d** TEM image, **e** HAADF image with corresponding elemental mapping, and **f** HRTEM image of MoS_2_–N–C_700_. **g** Schematic illustration of expanded interlayer spacing of MoS_2_ due to Mo–N bridging with N-doped carbon layer

The N-bridged MoS_2_/C heterostructures were synthesized via a surfactant-mediated strategy that combined precise coordination chemistry with an in situ conversion process (Fig. 1a). The synthesis begins with the confinement of a quaternary ammonium surfactant, cetyltrimethylammonium bromide (CTAB), within the dendritic mesopores of mesoporous SiO_2_ templates (designated as mSiO_2_@CTAB, Figs. S1 and S2). Subsequent introduction of molybdate anions induces electrostatic coordination with the positively charged quaternary ammonium headgroups, yielding mSiO_2_@CTAB-MoO_*x*_ composites. Notably, the mSiO_2_ template primarily acts as a nanoconfinement reactor rather than a rigid morphology-replication scaffold. During subsequent thermal treatment with thiourea, the MoO_*x*_ precursors are converted into MoS_2_ (Fig. S3), while the CTAB molecules undergo carbonization to form N-doped carbon layers. Concurrently, interfacial Mo–N bonds are formed between Mo species and nitrogen-containing carbon species, enabling intimate coupling between MoS_2_ and the carbon framework and guiding the formation of well-defined heterostructures. Meanwhile, the decomposition of surfactant species releases gaseous products, reconstructing the carbon framework and generating abundant mesopores throughout the composite.

To verify the carbon origin, mSiO_2_@CTAB was pyrolyzed under Ar atmosphere. A color change from white to black was observed, indicating the transformation of CTAB into amorphous carbon (Fig. S4). The carbonized product, named mSiO_2_@C, shows significant mass loss between 430 and 600 °C in thermogravimetric (TG) and differential TG (DTG) analyses (Fig. 1b), corresponding to the decomposition of CTAB-derived carbon species. The TG curve of mSiO_2_@CTAB under Ar shows gradual mass loss from 200 to 600 °C, primarily due to the decomposition of hexadecyl chains into carbon components. In contrast, pure CTAB exhibits a rapid decomposition even between 200 and 350 °C (Fig. S5), indicating that confinement within the mSiO_2_ framework enhances thermal stability and promotes carbon formation. Additionally, TG analysis of thiourea confirms its decomposition contributes additional carbonaceous species, which deposit onto the material to further stabilize the MoS_2_ structure (Fig. S6).

Field emission scanning electron microscopy (FESEM) images of the MoS_2_–N–C heterostructures reveal a porous architecture composed of interconnected irregular aggregates (Figs. 1c and S7). The seemingly fragmented morphology observed after template removal does not indicate structural collapse. Instead, it represents the expected porous inverse structure generated by hard-templating process. As the sulfurization temperature increases from 600 to 800 °C, the aggregates become progressively denser and the overall porosity gradually decreases, suggesting temperature-dependent densification of the carbon–MoS_2_ framework. Transmission electron microscopy (TEM) images of MoS_2_–N–C_700_ further confirm the porous and hierarchical structure and indicate a polycrystalline nature (Figs. 1d and S8). In contrast, commercial MoS_2_ exhibits a bulky morphology composed of heavily stacked multilayered domains (Fig. S9). Energy-dispersive X-ray spectroscopy (EDS) elemental mapping of MoS_2_–N–C_700_ reveals the uniform distribution of Mo, S, C, and N, confirming the nanoscale integration of nitrogen-doped carbon with MoS_2_ (Figs. 1e and S10). High-resolution TEM (HRTEM) image further reveals intimate contact between amorphous carbon layers and MoS_2_ nanodomains (Fig. 1f). The few-layered MoS_2_ displays an expanded interlayer spacing of 0.633 nm for MoS_2_, moderately larger than the 0.622 nm of commercial MoS_2_ (Fig. S11). This expansion confirms that the Mo–N bridging between the carbon layer and MoS_2_ creates a tensile effect, pulling Mo atoms toward the carbon side (Fig. 1g).

### Interfacial Mo–N Bridging between MoS_2_ and Carbon Layers

**Fig. 2:**
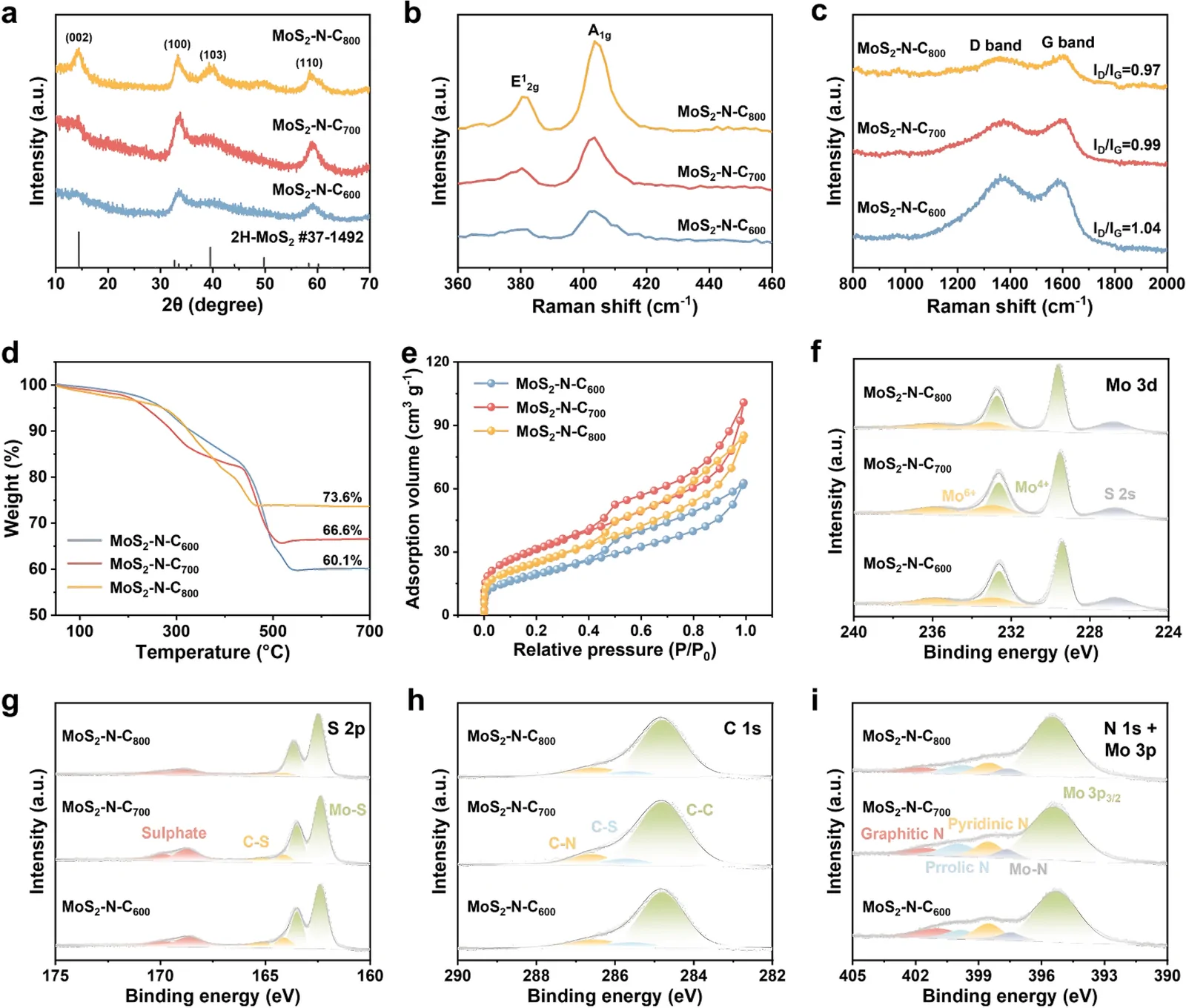
Compositional and chemical state analyses of the MoS_2_–N–C heterostructures synthesized at different sulfurization temperatures. **a** XRD patterns, **b, c** Raman spectra, **d** TG curves, **e** N_2_ absorption/desorption isotherms, **f–i** deconvoluted Mo 3*d*, S 2*p*, C 1*s,* and N 1*s* + Mo 3*p* XPS spectra, respectively

X-ray diffraction (XRD) analysis was conducted to investigate the effect of sulfurization temperature on the crystallinity of MoS_2_–N–C heterostructures (Fig. 2a). All samples display distinct diffraction peaks of the 2H–MoS_2_ phase, confirming the formation of a thermodynamically stable hexagonal polymorph. The peaks at approximately 13.9°, 33.3°, 39.7°, and 58.6° are indexed to the (002), (100), (103), and (110) crystal planes of 2H–MoS_2_ (PDF#37-1492), respectively [25]. Notably, the (002) peak shifts toward lower diffraction angles compared with bulk MoS_2_, indicating an expanded interlayer spacing. This observation is consistent with the TEM results and suggests the modulation effect of carbon layers on the layered structure of MoS_2_. As the sulfurization temperature increases from 600 to 800 °C, the intensity and sharpness of the (002) peak gradually increase, indicating improved crystallinity. In contrast, commercial MoS_2_ exhibits a much stronger and sharper (002) reflection, characteristic of highly crystalline bulk MoS_2_ (Fig. S12).

Raman spectroscopy further confirms the structural evolution of the MoS_2_–N–C heterostructures. As shown in Fig. 2b, two characteristic vibrational modes at 380.4 cm^−1^ (E^1^_2g_) and 403.5 cm^−1^ (A_1g_) are observed, confirming the formation of few-layer 2H–MoS_2_. The intensities of both peaks increase with increasing sulfurization temperature, indicating enhanced crystallinity. In contrast, the D and G bands associated with the carbon component gradually weaken with increasing temperature. The corresponding decrease in the *I*_D_/*I*_G_ ratio from 1.04 to 0.99 and 0.97 (Fig. 2c) suggests a reduction in defect density and an increase in graphitization degree of the carbon matrix. Meanwhile, the relative intensity variation between MoS_2_ and carbon bands indicates a progressive increase in the MoS_2_ fraction at higher temperatures (Fig. S13) [13].

TG analysis provides quantitative data on the MoS_2_ content in the MoS_2_–N–C heterostructures. MoS_2_–N–C_600_, MoS_2_–N–C_700_, and MoS_2_–N–C_800_ show weight retention values of 60.1%, 66.6%, and 73.6%, respectively, after calcination in air, corresponding to 66.8%, 74.1%, and 81.8% MoS_2_ content by weight (Fig. 2d). These results align with XRD and Raman analysis, indicating that higher sulfurization temperatures resulted in a higher MoS_2_ content in the heterostructures. The carbon component is derived from the in situ carbonization of the alkyl chains of CTAB surfactant and plays a crucial role in constructing the overall MoS_2_–N–C nanostructure. The carbon matrix primarily serves as a conductive and structural backbone, improving electrical conductivity and buffering the volume variation of MoS_2_ during cycling. Nitrogen adsorption–desorption isotherms confirm the presence of a well-defined mesoporous structure in all the materials, as indicated by type-IV isotherms (Figs. 2e and S14). The specific surface areas (SSAs) of MoS_2_–N–C_600_, MoS_2_–N–C_700_, and MoS_2_–N–C_800_ are 70.9, 112.9, and 90.9 m^2^ g^−1^, respectively. MoS_2_–N–C_700_ has the highest SSA with a most probable pore size of ~ 2.0 nm, which is favorable for Li^+^ diffusion and electrolyte accessibility.

X-ray photoelectron spectroscopy (XPS) was conducted to investigate the surface chemical states of these MoS_2_–N–C heterostructures (Fig. S15). The Mo 3*d* spectra display two main peaks at 229.5 and 232.6 eV, corresponding to 3*d*_3/2_ and 3*d*_1/2_ signals of Mo^4+^ species in MoS_2_, along with two smaller peaks at 232.8 and 235.9 eV for Mo^6+^ species due to partial surface oxidation (Fig. 2f) [26]. The S 2*p* spectra exhibit Mo–S bonds at 162.4 and 163.5 eV, C–S bonds at 164.1 and 165.2 eV, and oxidized sulfur species at 168.7 and 169.8 eV (Fig. 2g) [27, 28]. The relative proportion of Mo–S species increases with sulfurization temperature, consistent with Raman results (Table S1). The C 1*s* spectra reveal three peaks at 284.8, 285.7, and 286.6 eV, attributed to C–C, C–N, and C–S bonds, respectively (Fig. 2h) [29]. The C–S species might come from the thiourea-derived carbon supports. As shown in the N 1*s* spectra, four distinct peaks are observed at 397.6, 398.5, 399.8, and 401.3 eV, corresponding to Mo–N, pyridinic N, pyrrolic N, and graphitic N species, respectively (Fig. 2i) [30]. Notably, the presence of Mo–N bonding is further confirmed by the Fourier-transformed (FT) extended X-ray absorption fine structure (EXAFS) spectrum of Mo K-edge, which exhibits a characteristic first-shell scattering contribution associated with Mo–N coordination (Fig. S16) [25]. These results provide direct evidence for the nitrogen-bridged interfacial bonding between MoS_2_ and the carbon layer. Among the nitrogen configurations, pyridinic N and pyrrolic N are the dominant species, which are well known to introduce abundant structural defects and active sites within the carbon matrix, thereby enhancing pseudocapacitive contributions and facilitating Li^+^ diffusion. In contrast, graphitic N content increases with temperature, reflecting thermally driven conversion of less stable nitrogen species into more stable configurations, which contributes to improved electronic conductivity of the carbon framework (Table S2). The existence of C–N species in both the C 1*s* and N 1*s* spectra confirms the successful transformation of CTAB into an N-doped carbon layer, which was chemically linked to MoS_2_ via Mo–N bonds. To further verify the origin of Mo–N bonding, a nitrogen-free MoS_2_ control sample (denoted as Pristine MoS_2_) was synthesized under identical sulfurization conditions without CTAB-capped mSiO_2_ templates. This sample exhibits a bulk-like aggregated morphology without internal porosity (Fig. S17). More importantly, no N 1*s* signal or Mo–N bonding is detected in XPS spectra (Fig. S18), confirming that the Mo–N bonds originate from the CTAB-derived N-doped carbon framework.

Electron paramagnetic resonance (EPR) spectra exhibit a weakening signal at *g* = 2.003 (Fig. S19), suggesting the existence of sulfur vacancies [15, 31]. These vacancies are likely associated with interfacial Mo–N coordination, which modulates the local electronic structure of MoS_2_ and alters the defect distribution. The gradual decrease in signal intensity with increasing temperature indicates that Mo–N coordination effectively tunes the electronic environment and stabilizes defect configurations within the heterostructure.

### Electrochemical Performance for Lithium-Ion Batteries

**Fig. 3:**
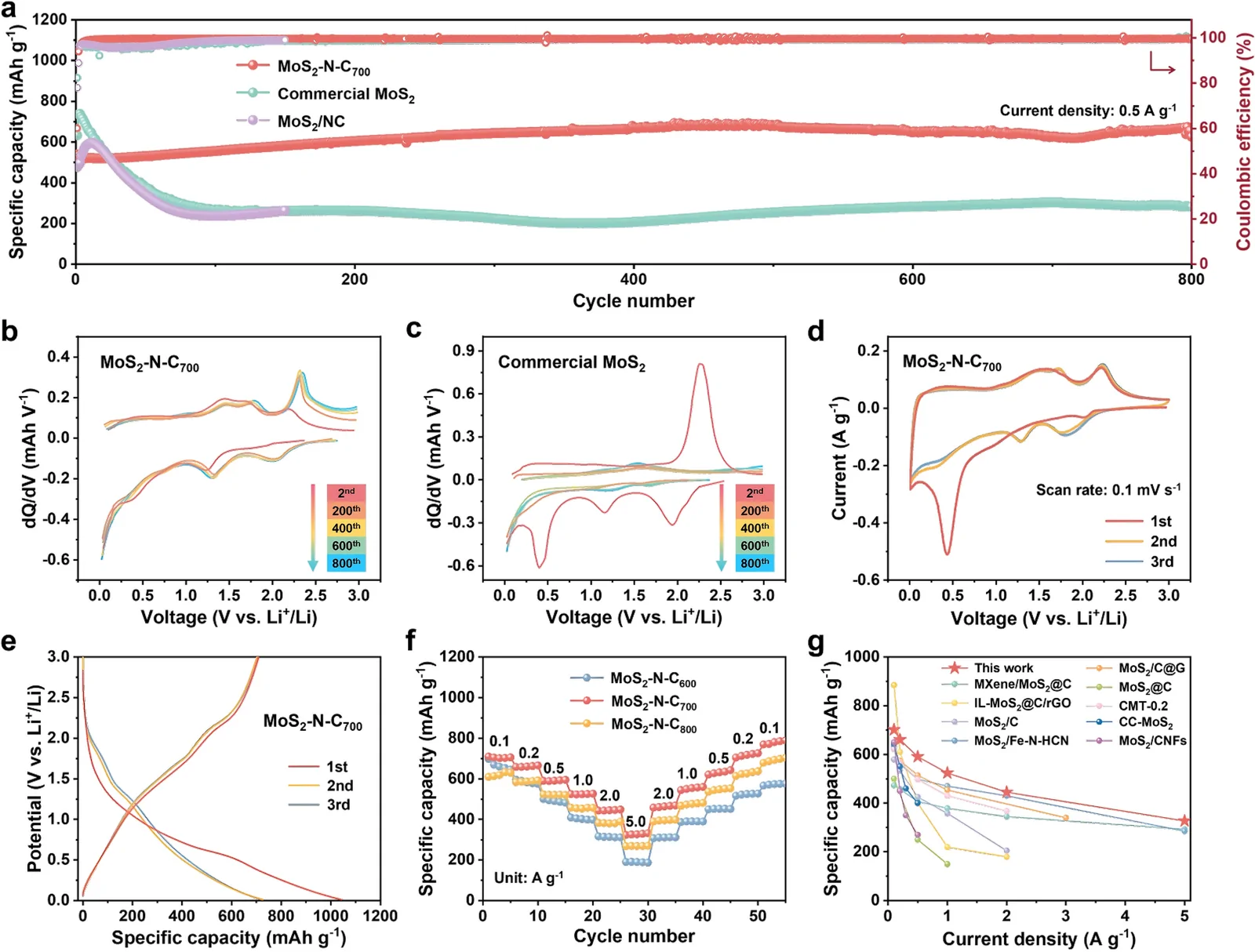
Electrochemical performance of the MoS_2_–N–C anodes for LIBs. **a** Cycling stability at 0.5 A g^−1^. **b, c** d*Q*/d*V* curves of MoS_2_–N–C_700_ and commercial MoS_2_ at different cycles. **d** First three CV curves at 0.1 mV s^−1^ and **e** first three GCD profiles at 0.1 A g^−1^ of MoS_2_–N–C_700_. **f** Rate capacities and **g** comparison of rate performances between MoS_2_–N–C_700_ and recently reported MoS_2_/C composites

The MoS_2_–N–C heterostructures were systematically evaluated as anode materials for lithium-ion batteries (LIBs) at the mass loading of 1.1–1.3 mg cm^−2^. As shown in Fig. 3a, the MoS_2_–N–C_700_ electrode delivers an initial discharge capacity of 539.2 mAh g^−1^ at 0.5 A g^−1^, and gradually increases to 625.9 mAh g^−1^ after 800 cycles, accompanied by nearly 100% Coulombic efficiency. This capacity increase indicates excellent structural reversibility without an increase in defect density (Fig. S20) but progressive activation of the porous structure and additional redox sites during cycling (Figs. S21 and S22). The integrity of the interfacial Mo–N bridges in MoS_2_–N–C_700_ is further confirmed by ex situ XPS analysis after cycling (Fig. S23). The cycling performance of MoS_2_–N–C_700_ surpasses that of most previously reported MoS_2_/C nanocomposites (Table S4). In contrast, the MoS_2_–N–C_600_ electrode maintains the lowest reversible capacity (~ 400 mAh g^−1^) throughout the cycling test (Fig. S24), while the MoS_2_–N–C_800_ electrode shows obvious capacity decay after 500 cycles (Fig. S25). These distinct electrochemical behaviors highlight the critical balance among MoS_2_ crystallinity, interfacial Mo–N coupling strength, and carbon framework integrity in achieving high-capacity and long-life cycling performance. The Mo–N bridges function as both efficient electron-transfer pathways and robust structural anchors that buffer volume changes during cycling. Their effectiveness strongly depends on the integrity of the carbon framework (Fig. 2d). Although MoS_2_–N–C_600_ possesses the highest carbon fraction (33.2 wt%), its insufficient MoS_2_ crystallization limits the formation of effective heterointerfaces. In contrast, MoS_2_–N–C_700_ achieves an optimized balance between carbon content (25.9 wt%) and interfacial Mo–N coupling, enabling both efficient charge transport and structural buffering. For MoS_2_–N–C_800_, despite its highly crystallinity, the substantially reduced carbon framework (18.2 wt%) weakens interfacial anchoring and structural stability, resulting in accelerated capacity fading during prolonged cycling.

In contrast, commercial MoS_2_ suffers from rapid capacity decay during the first 50 cycles, ultimately retaining only 282.8 mAh g^−1^ (44.6% retention) after 800 cycles. For comparison, a physical nitrogen-doped carbon and MoS_2_ hybrid (MoS_2_/NC) without interfacial Mo–N bridging was also prepared (more details in Supporting Information). MoS_2_/NC exhibits a much lower specific surface area (66.8 m^2^ g^−1^) than MoS_2_–N–C_700_ (112.9 m^2^ g^−1^) (Fig. S26) and lacks a well-defined porous structure (Fig. S27), demonstrating the advantage of the proposed synthetic strategy. As a result, MoS_2_/NC displays poor cycle stability with only 263.6 mAh g^−1^ (55.2% retention) after 150 cycles. These results highlight the significant role of interfacial Mo–N bridging in enhancing the cycling stability of MoS_2_. Moreover, the MoS_2_–N–C_700_ anode also displays decent cycling stability at 2 A g^−1^ for 1000 cycles (Fig. S28), with Coulombic efficiency close to 100% throughout cycling.

Differential capacity analysis (d*Q*/d*V*) further confirmed the superior reversibility of the MoS_2_–N–C electrodes. The MoS_2_–N–C_700_ anode shows highly overlapped redox peaks from the 2nd to the 800th cycle, indicating excellent structural stability and highly reversible phase transitions during cycling (Figs. 3b and S29). Conversely, both MoS_2_/NC and commercial MoS_2_ show significant redox peak shifting and intensity fading during cycling, suggesting progressive electrode polarization and irreversible structural degradation (Figs. 3c and S30).

Cyclic voltammetry (CV) tests were employed to gain further insights into the electrochemical reversibility and lithiation/delithiation mechanisms (Figs. 3d and S31). The first cathodic scan for MoS_2_–N–C_700_ shows a weak peak at ~ 0.90 V and a sharp reduction peak at ~ 0.43 V, corresponding to Li^+^ intercalation into MoS_2_ layers and the conversion of Li_*x*_MoS_2_ into metallic Mo and Li_2_S, respectively. Subsequent anodic scan displays two distinct oxidation peaks at ~ 1.59 and ~ 2.22 V, associated with the reformation of MoS_2_ from metallic Mo and the oxidation of Li_2_S to element sulfur, respectively [5, 32]. In the following cycles, the CV curves stabilize and exhibit two well-defined cathodic peaks at ~ 1.75 and ~ 1.28 V, attributed to the reduction of S to Li_2_S and the formation of Li_x_MoS_2_, respectively, along with two stable anodic peaks consistent with the first cycle [33]. For MoS_2_/NC and commercial MoS_2_, a prominent peak occurs at ~ 0.90 V in the first cathodic sweep, suggesting typical Li^+^ intercalation into the MoS_2_ layers but lacking the stable and well-defined redox features observed for MoS_2_–N–C_700_ (Figs. S32 and S33). The anodic peak associated with Mo reoxidation is significantly suppressed, confirming inferior reversibility and structural instability. Among all samples, MoS_2_–N–C_700_ exhibits the highest specific capacity, with discharge and charge capacities of 1044.7 and 709.2 mAh g^−1^, respectively (Figs. 3e and S34, S35). MoS_2_–N–C_700_ exhibits an initial Coulombic efficiency of approximately 68% in LIB half-cells. The irreversible capacity loss is primarily attributed to solid–electrolyte interphase formation and surface defect-related Li^+^ trapping during the initial lithiation process, which is typical for porous nanostructured conversion/intercalation-type MoS_2_-based anodes.

The MoS_2_–N–C_700_ anode also demonstrates outstanding rate capability (Fig. 3f). It delivers reversible capacities of 709.2, 660.0, 590.6, 523.6, 444.8, and 326.8 mAh g^−1^ at current densities of 0.1, 0.2, 0.5, 1.0, 2.0, and 5.0 A g^−1^, respectively. When the current density is returned to 0.1 A g^−1^, the capacity recovers to 788.4 mAh g^−1^, confirming excellent electrochemical reversibility and robust structural stability. Across all current densities, MoS_2_–N–C_700_ consistently outperforms its counterparts synthesized at other sulfurization temperatures. More importantly, its rate performance surpasses that of most previously reported MoS_2_/C nanocomposites [24, 29, 32, 34–39], highlighting the effectiveness of the interfacial Mo–N bridging strategy for constructing high-performance heterostructured electrodes (Fig. 3g). Notably, when the mass loading is increased to ~ 2.9 mg cm^−2^, MoS_2_–N–C_700_ maintains outstanding rate capability with reversible capacities comparable to those obtained at a lower mass loading (Fig. S36). It also exhibits stable cycling with a high capacity of ~ 600 mAh g^−1^ at 0.5 A g^−1^ over 120 cycles. In contrast, commercial MoS_2_ suffers from a dramatic capacity drop at high rates due to its poor intrinsic conductivity and structural vulnerability, with specific capacities of only 276.7 and 116.4 mAh g^−1^ at 2.0 and 5.0 A g^−1^, respectively (Fig. S37). Similarly, MoS_2_/NC exhibits inferior rate capability with specific capacities of only 406.1 and 241.5 mAh g^−1^ at 2.0 and 5.0 A g^−1^, respectively.

### Structure Evolution During Long-Term Cycling

**Fig. 4:**
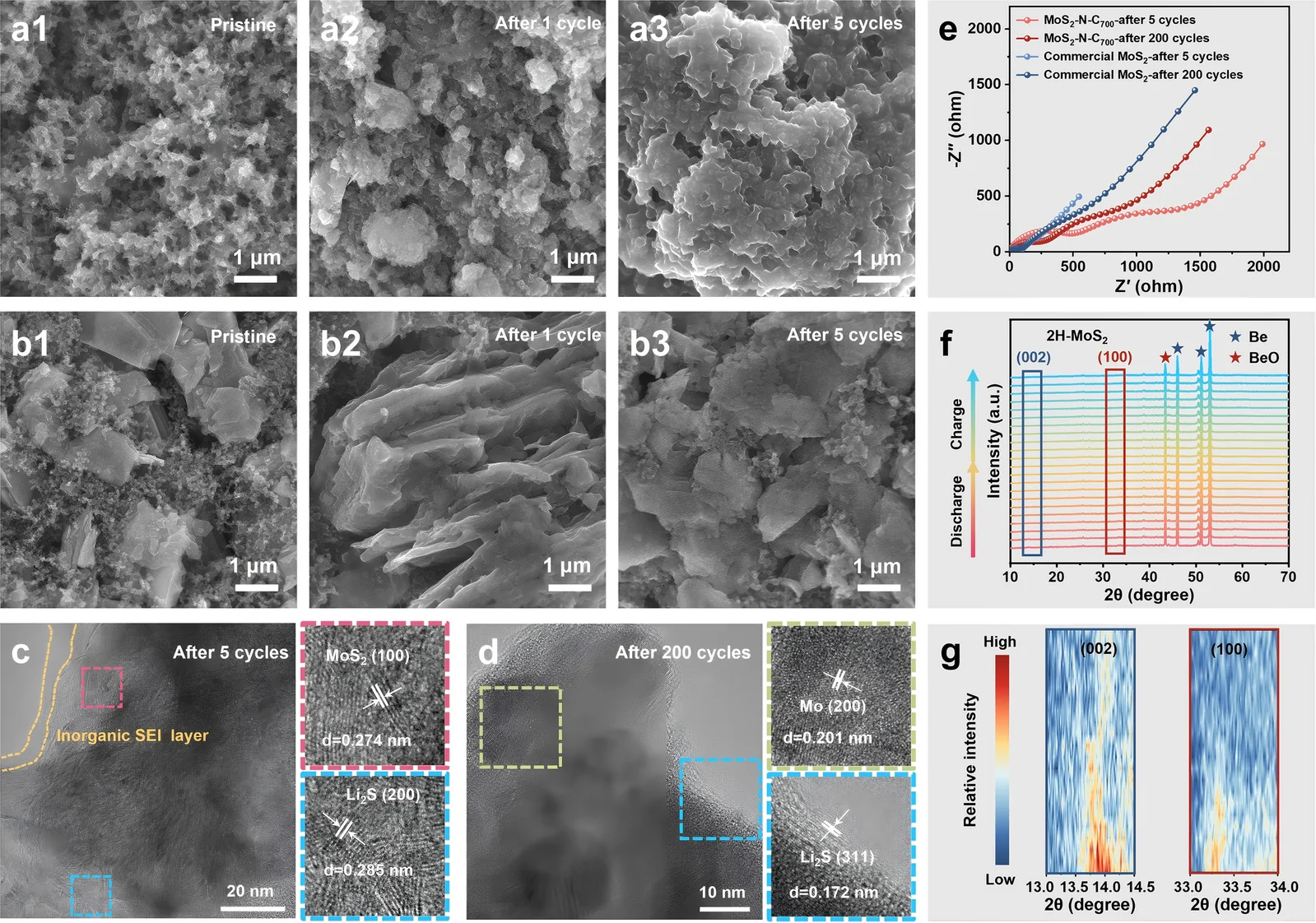
Structure evolution and electrochemical behavior of the MoS_2_–N–C and commercial MoS_2_ anodes during cycling in LIBs. **a1–a3** FESEM images of the pristine and post-cycled MoS_2_–N–C_700_ electrodes. **b1–b3** FESEM images of the pristine and post-cycled commercial MoS_2_ electrode. **c, d** HRTEM images of the post-cycled MoS_2_–N–C_700_ electrodes. **e** Nyquist plots of the post-cycled MoS_2_–N–C_700_ and commercial MoS_2_ electrodes. **f** In situ XRD patterns and **g** enlarged contour plots of characteristic (002) and (100) peaks of MoS_2_–N–C_700_ during the first GCD cycle

The morphological evolution of the electrodes during cycling was investigated. The pristine MoS_2_–N–C_700_ electrode exhibits a highly porous interconnected nanostructure composed of uniformly distributed nanoparticle agglomerates (Fig. 4a). In contrast, the pristine commercial MoS_2_ electrode consists of large micron-sized layered flakes with loose stacking and obvious interparticle gaps (Fig. 4b). During cycling, the MoS_2_–N–C_700_ electrode undergoes only slight morphological adjustment, while its porous framework remains largely preserved and the surface becomes more uniform. Such structural robustness effectively accommodates the repeated Li^+^ insertion/extraction processes and preserves electrode integrity. In contrast, the commercial MoS_2_ electrode experiences severe structural degradation even after the first cycle. The layered flakes become heavily stacked and distorted, and the originally well-defined flake boundaries gradually disappear due to pronounced exfoliation induced by Li^+^ intercalation. After five cycles, the structure collapses into aggregated fine particles, leading to serious deterioration of electrode integrity and cycling stability.

HRTEM images further confirm the structural stability of the MoS_2_–N–C_700_ electrode. After five cycles, a uniform inorganic solid–electrolyte interphase (SEI) layer with a thickness of only a few nanometers is observed on the electrode surface (Fig. 4c). Clear lattice fringes with spacings of 0.274 and 0.285 nm are attributed to the (100) plane of MoS_2_ and (200) plane of Li_2_S, respectively, suggesting the coexistence of reversible conversion and intercalation processes. Even after 200 cycles, distinct diffraction patterns can still be indexed to Mo (200) and Li_2_S (311), with spacings of 0.201 and 0.172 nm, respectively, confirming the structural durability of the active material (Fig. 4d). XPS analysis of the MoS_2_–N–C_700_ electrode discharged to 0.01 V reveals a distinct Mo–N peak at 397.3 eV (Fig. S38), indicating that the interfacial coupling between Mo species and N-doped carbon framework remains intact even after deep conversion reactions.

Electrochemical impedance spectra (EIS) reveal that the MoS_2_–N–C_700_ electrode maintains significantly lower impedance than both commercial MoS_2_ and the MoS_2_/NC throughout cycling (Figs. 4e and S39–S41). Specifically, the MoS_2_–N–C_700_ electrode exhibits charge-transfer resistances (*R*_ct_) of 77.1 and 229.1 Ω after 5 and 200 cycles, respectively, whereas commercial MoS_2_ shows much higher values of 1605.0 and 312.2 Ω (Table S3). The MoS_2_/NC control sample shows a reduced *R*_ct_ of 164.6 Ω, confirming that the introduction of a conductive carbon framework can improve charge transfer to some extent. However, in the absence of chemical interfacial bonding, the electronic interaction between MoS_2_ and carbon remains limited. These results further demonstrate that the N-bridged heterostructure not only stabilizes electrode morphology but also sustains efficient charge-transfer kinetics during long-term cycling, in stark contrast to the rapid degradation observed for commercial MoS_2_.

In situ XRD patterns were further employed to investigate the phase evolution of the MoS_2_–N–C_700_ electrode during the first GCD cycle (Fig. 4f). The contour color transition from red to blue corresponds to different charge/discharge states. As shown in the enlarged contour plots (Fig. 4g), during discharge, the (002) peak gradually shifts toward lower angles, indicating Li^+^ intercalation into the MoS_2_ interlayers and the associated expansion of interlayer spacing. During the subsequent charging process, the peak gradually shifts back to its original position, demonstrating highly reversible intercalation behavior. Meanwhile, the intensity of the (100) peak continuously decreases and eventually disappears during discharge, suggesting the conversion of MoS_2_ into metallic Mo and Li_2_S. During charging, the (002) peak reappears, confirming the excellent reversibility of the conversion reaction.

### Lithium-Ion-Transport Kinetics Analysis

**Fig. 5:**
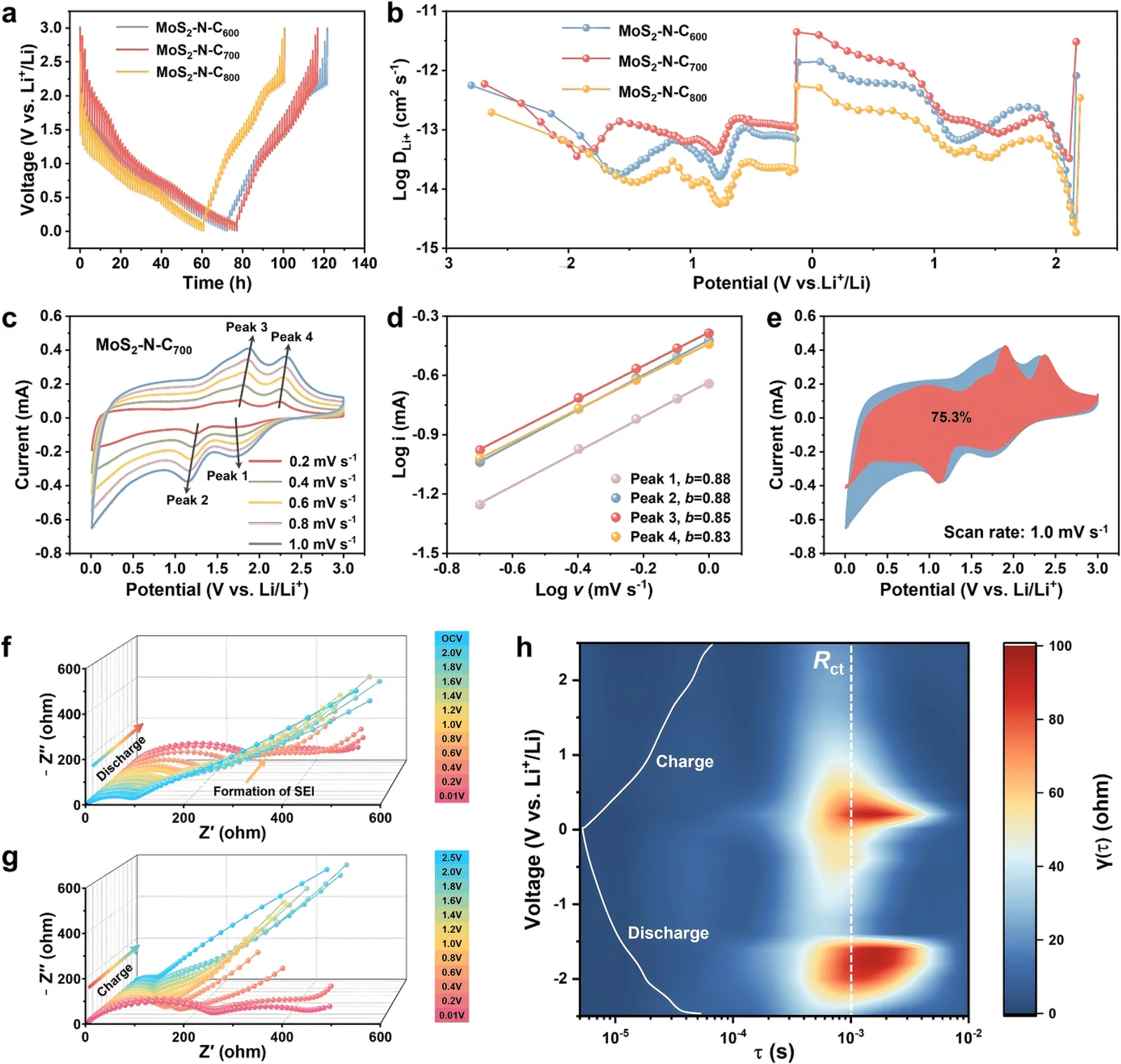
Electrochemical kinetic analysis of the MoS_2_–N–C anodes for LIBs. **a** GITT curves after five GCD cycles. **b** Li^+^ diffusion coefficients derived from GITT curves of MoS_2_–N–C_600_, MoS_2_–N–C_700_, and MoS_2_–N–C_800_. **c** CV curves at scan rates from 0.2 to 1.0 mV s^−1^, **d** linear fitting of the logarithmic relationship between peak current (log *i*) and scan rate (log *v*), **e** pseudocapacitive contribution at 1.0 mV s^−1^, **f, g** In situ EIS spectra during discharge and charge, and **h** DRT contour plot of charge-transfer process (*R*_ct_) during the 6th charge–discharge cycle of MoS_2_–N–C_700_ (the negative voltage represents the discharging process)

Galvanostatic intermittent titration technique (GITT) was employed to investigate the Li^+^ diffusion kinetics of the MoS_2_–N–C heterostructures (Fig. 5a). The calculated Li^+^ diffusion coefficients (*D*_Li+_) indicate that MoS_2_–N–C_700_ exhibits the highest diffusion coefficient, reaching up to 1.64 × 10^–12^ cm^2^ s^−1^, indicative of highly efficient ion-transport pathways (Fig. 5b). Among all MoS_2_–N–C heterostructures, MoS_2_–N–C_700_ consistently displays higher *D*_Li+_ values during both discharge and charge processes. CV measurements at various scan rates further confirm the superior electrochemical kinetics and reversibility of the MoS_2_–N–C materials, with well-defined and symmetric redox peaks (Fig. 5c). The corresponding log(*i*)–log(*v*) plots yield *b*-values of 0.83, 0.88, 0.85, and 0.87 for the four redox peaks of MoS_2_–N–C_700_, indicating a dominant pseudocapacitive charge-storage behavior (Fig. 5d) [40, 41]. The specific pseudocapacitive contribution increases with scan rate, reaching 64.9%, 75.3%, and 70.0% for MoS_2_–N–C_600_, MoS_2_–N–C_700_, and MoS_2_–N–C_800_, respectively, at 1.0 mV s^−1^ (Figs. 5e and S42–S44). In contrast, MoS_2_/NC exhibits a much lower value of 40.9% (Fig. S45) than MoS_2_–N–C_700_, highlighting the critical roles of the N-doped carbon matrix, interfacial Mo–N coupling, and porous architecture for fast pseudocapacitive Li^+^ storage. These results confirm the dominance of fast surface-controlled charge-transfer kinetics in the MoS_2_–N–C heterostructures.

In situ EIS tests were conducted to investigate the dynamic electrochemical behavior of MoS_2_–N–C_700_ after activation at 0.1 A g^−1^ for five cycles (Figs. 5f, g and S46–S47). The *R*_ct_ value gradually increases during discharging from 3.0 to 0.01 V, followed by a decrease during charging, ultimately reaching 47.3 Ω. Notably, a distinct change in the Nyquist plots appears at approximately 0.8 V during lithiation, corresponding to the formation of a robust SEI layer. Distribution of relaxation times (DRT) plots obtained from the in situ EIS data further corroborates this evolution process (Figs. 5h and S50–S52) [42]. Moreover, the *D*_Li+_ of MoS_2_–N–C_700_ reaches a peak value of 5.38 × 10^–12^ cm^2^ s^−1^ during cycling, surpassing that of the other counterparts. Such outstanding kinetic behavior is attributed to the synergistic structural features of the heterostructures, including nitrogen-doped carbon layers that accelerate electron transfer through interfacial Mo–N coupling, expanded layer spacings of MoS_2_ that facilitate rapid Li⁺ diffusion, and porous morphology that endows structural robustness.

To validate the generality of the surfactant-mediated strategy, it was further extended to the WS_2_ system. The XRD pattern of the as-synthesized WS_2_–N–C_700_ sample (Fig. S53) matches well with hexagonal WS_2_ (PDF#87-2417). Notably, the barely discernible (002) diffraction peak at about 14.5° indicates suppressed layer stacking, confirming the effective nanoconfinement imposed by the mSiO_2_ template. This is consistent with the structural characteristics observed in the MoS_2_ system. SEM analysis (Fig. S54) further reveals that the WS_2_–N–C_700_ sample exhibits a similar interconnected porous architecture to that of the MoS_2_–N–C_700_ heterostructures. When applied as an anode material for LIBs, the WS_2_–N–C_700_ electrode exhibits a reversible intercalation-conversion Li^+^ storage process and excellent cycling stability (Fig. S55). It delivers a reversible capacity of 539.7 mAh g^−1^ at 0.1 A g^−1^, exceeding the theoretical capacity of bulk WS_2_ (~ 432 mAh g^−1^). Furthermore, the electrode exhibits exceptional cycling stability at 0.5 A g^−1^, maintaining nearly 100% capacity retention after 300 cycles. These results collectively support that the proposed mesopore-confined, surfactant-mediated strategy can serve as a generalizable platform for synthesizing N-bridged LTMD/C heterostructures beyond MoS_2_.

### Device-Level Lithium-Ion Supercapacitor Performance

**Fig. 6:**
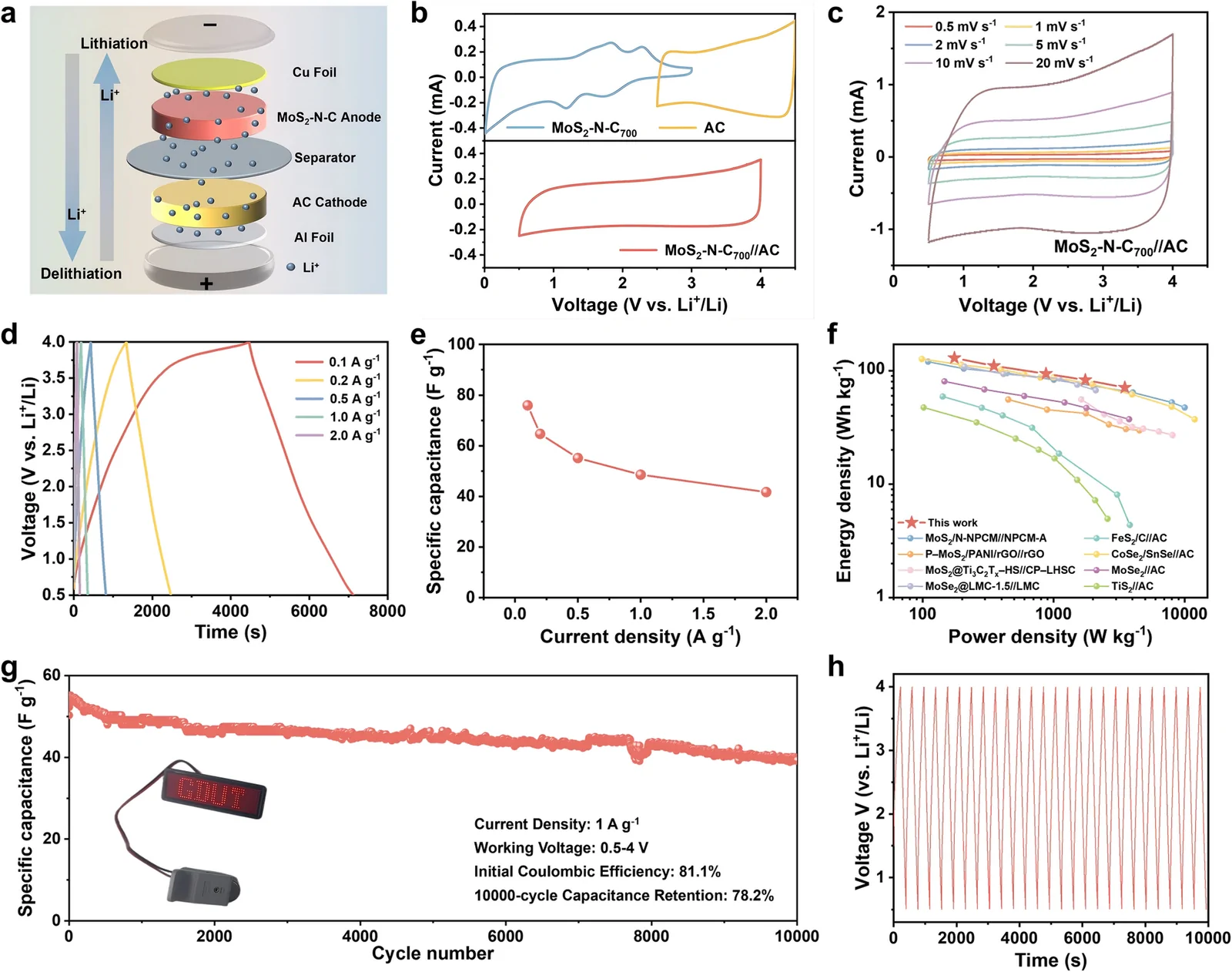
Electrochemical evaluation of lithium-ion supercapacitor (LISC) assembled with the MoS_2_–N–C_700_ heterostructure anode and activated carbon (AC) cathode. **a** Schematic of the LISC configuration. **b** Operating windows of MoS_2_–N–C_700_ anode, AC cathode and MoS_2_–N–C_700_//AC device. **c** CV curves at different scan rates. **d** GCD curves at different current densities. **e** Specific capacitances of MoS_2_–N–C_700_//AC device at different current densities. **f** Ragone plot comparing the energy and power densities of MoS_2_–N–C_700_//AC device with reported transition-metal dichalcogenide (TMD)-based LISCs. **g** Cycling stability of the MoS_2_–N–C_700_//AC device at 1 A g^−1^, with the inset showing LED illumination. **h** GCD curves during the initial 10,000 s

Benefiting from the excellent structural stability and surface-controlled charge-storage behavior, MoS_2_–N–C_700_ emerges as a promising anode for high-performance lithium-ion supercapacitors (LISCs). A full device was constructed by using MoS_2_–N–C_700_ as the anode and commercial activated carbon (AC) as the cathode (Fig. 6a). The CV curves of the individual electrodes exhibit distinct electrochemical behaviors (Fig. 6b). The MoS_2_–N–C_700_ anode exhibits broad redox peaks associated with reversible faradaic reactions, while the AC cathode presents a nearly rectangular profile typical of electrical double-layer capacitive (EDLC) behavior [43, 44]. When assembled, the MoS_2_–N–C_700_//AC LISC exhibits a quasi-rectangular CV profile across a broad voltage window of 0.5–4.0 V, integrating both faradaic and capacitive processes (Fig. 6c). The operating voltage window of MoS_2_–N–C_700_//AC is carefully selected based on the stable potential ranges of both electrodes. Across scan rates from 0.5 to 20 mV s^−1^, the device retains its capacitive shape, highlighting its excellent reversibility and high-rate adaptability.

The GCD curves of MoS_2_–N–C_700_//AC further confirm the capacitive nature, showing nearly symmetric triangular shapes with minimal *iR* drop across different current densities (Fig. 6d). The device delivers specific capacitances of 75.9, 64.7, 55.1, 48.6, and 41.7 F g^−1^ at current densities of 0.1, 0.2, 0.5, 1.0, and 2.0 A g^−1^, respectively, suggesting its outstanding rate capability (Fig. 6e). The energy and power outputs are assessed by a Ragone plot (Fig. 6f). The MoS_2_–N–C_700_//AC LISC delivers a high energy density of 175 Wh kg^−1^ at a power density of 129.2 W kg^−1^ and maintains 80.0 Wh kg^−1^ even at 3500 W kg^−1^. The energy density outperforms most reported transition-metal dichalcogenide (TMD)-based LISCs and traditional carbon-based anodes (Table S5) [26, 40, 45–50].

The device also possesses remarkable long-term cycling stability. At 1 A g^−1^, it retains 78.2% of its initial capacitance after 10,000 cycles, with an initial Coulombic efficiency of 81.1% (Fig. 6g). The ability to power an LED further verifies its stable energy output and practical applicability. Moreover, stable operation is maintained across the full voltage window (Fig. 6h). Overall, the MoS_2_–N–C heterostructure enables a LISC device that simultaneously achieves high energy density, superior power density, and outstanding durability, positioning it as a promising candidate for advanced energy storage applications.

The outstanding electrochemical performance originates from the rationally designed heterostructure. In this system, a surfactant-mediated synthesis strategy is employed to construct N-bridged MoS_2_/C heterostructures, in which the alkyl chains of CTAB undergo in situ carbonization to generate N-doped carbon layers. Meanwhile, CTAB decomposition releases gaseous species that reconstruct the carbon framework and produce abundant mesopores. More importantly, Mo–N bonds are formed at the MoS_2_/C interfaces, enabling strong interfacial coupling and stabilizing the integrated porous structure. The N-doped carbon matrix serves as a conductive and robust framework, facilitating electron transport and buffering volume variation of MoS_2_ during cycling. The MoS_2_ component functions as the primary active phase for Li^+^ storage. In parallel, the hierarchical porous architecture suppresses MoS_2_ restacking, exposes abundant active edge sites, enhances electrolyte accessibility, and accommodates structural strain during repeated cycling.

## Conclusions

In summary, we have demonstrated a quaternary ammonium surfactant-mediated strategy to construct porous MoS_2_/C heterostructures with N-doped carbon layers chemically linked to MoS_2_ via Mo–N bridging. This chemically integrated architecture effectively strengthens interfacial stability, expands the interlayer spacing of MoS_2_, and facilitates fast electron/ion transport. The optimized heterostructure delivers outstanding electrochemical performance as an anode for lithium-ion batteries, including excellent cycling stability retaining ~ 100% capacity after 800 cycles at 0.5 A g^−1^, and superior rate capability. Beyond batteries, it also demonstrates excellent performance in lithium-ion supercapacitors, achieving stable cycling over 10,000 cycles. This work provides a versatile design strategy for constructing chemically integrated heterostructure electrodes for high-performance and durable electrochemical energy storage devices.

## Supplementary Information

Below is the link to the electronic supplementary material.

Supplementary file1 (DOCX 11128 KB)

## References

[1] Z. Zhu, T. Jiang, M. Ali, Y. Meng, Y. Jin et al., Rechargeable batteries for grid scale energy storage. Chem. Rev. **122**(22), 16610–16751 (2022). 10.1021/acs.chemrev.2c00289 36150378 10.1021/acs.chemrev.2c00289

[2] Y. Wang, R. Wang, K. Tanaka, P. Ciais, J. Penuelas et al., Accelerating the energy transition towards photovoltaic and wind in China. Nature **619**(7971), 761–767 (2023). 10.1038/s41586-023-06180-8 37495878 10.1038/s41586-023-06180-8 PMC10371865

[3] Y. Dou, Z. Liu, L. Zhao, J. Zhang, F. Meng et al., Constructing double heterojunctions on 1T/2H–MoS_2_@Co_3_S_4_ electrocatalysts for regulating Li_2_O_2_ formation in lithium-oxygen batteries. Nano-Micro Lett. **18**(1), 51 (2025). 10.1007/s40820-025-01895-x 10.1007/s40820-025-01895-x PMC12401821 40888861

[4] S. Roy, A. Joseph, X. Zhang, S. Bhattacharyya, A.B. Puthirath et al., Engineered two-dimensional transition metal dichalcogenides for energy conversion and storage. Chem. Rev. **124**(16), 9376–9456 (2024). 10.1021/acs.chemrev.3c00937 39042038 10.1021/acs.chemrev.3c00937

[5] B. Chen, D. Chao, E. Liu, M. Jaroniec, N. Zhao et al., Transition metal dichalcogenides for alkali metal ion batteries: engineering strategies at the atomic level. Energy Environ. Sci. **13**(4), 1096–1131 (2020). 10.1039/c9ee03549d

[6] Y. Wang, S. Sarkar, H. Yan, M. Chhowalla, Critical challenges in the development of electronics based on two-dimensional transition metal dichalcogenides. Nat. Electron. **7**(8), 638–645 (2024). 10.1038/s41928-024-01210-3

[7] M. Liang, H. Zhang, B. Chen, X. Meng, J. Zhou et al., A universal cross-synthetic strategy for sub-10 nm metal-based composites with excellent ion storage kinetics. Adv. Mater. **35**(52), e2307209 (2023). 10.1002/adma.202307209 37729880 10.1002/adma.202307209

[8] Z. Li, M. Han, Y. Zhang, F. Yuan, Y. Fu et al., Single-layered MoS_2_ fabricated by charge-driven interlayer expansion for superior lithium/sodium/potassium-ion-battery anodes. Adv. Sci. **10**(15), 2207234 (2023). 10.1002/advs.202207234 10.1002/advs.202207234 PMC10214217 36950770

[9] Z. Li, M. Han, J. Wang, L. Zhang, P. Yu et al., Superparamagnetic Fe conversion induces MoS_2_ fast ion transport in wide-temperature-range sodium-ion batteries. Adv. Funct. Mater. **34**(41), 2404263 (2024). 10.1002/adfm.202404263

[10] S. Liu, K. Jia, J. Yang, S. He, Z. Liu et al., Encapsulating flower-like MoS_2_ nanosheets into interlayer of nitrogen-doped graphene for high-performance lithium-ion storage. Chem. Eng. J. **475**, 146181 (2023). 10.1016/j.cej.2023.146181

[11] B. Cheng, Y. He, C. Li, H. Liu, B. Sun et al., Space-confined MoS_2_ in gradient-structured carbon spheres for ultra-stable sodium-ion storage. Adv. Funct. Mater. **36**(15), e16499 (2026). 10.1002/adfm.202516499

[12] W. Zhu, J. Zhao, X. Tao, MoS_2_–carbon based nanocomposites as anodes for lithium-ion batteries: a review. J. Energy Storage **84**, 110934 (2024). 10.1016/j.est.2024.110934

[13] L. Liu, W. Du, Q. Zhang, H. Jiang, Y. Zhang et al., Constructing hollow flower-like molybdenum disulfide nanospheres/carbon nanospheres as anode with enhanced diffusion kinetics for lithium storage. Adv. Compos. Hybrid Mater. **7**(6), 195 (2024). 10.1007/s42114-024-01029-8

[14] K.Y. Jang, Y.A. Lee, S. Lim, S. Park, K.-N. Jung et al., Enhancing lithium-ion battery kinetics and stability leveraging hybrid 1T/2H MoS_2_–graphene heterostructures. Chem. Eng. J. **520**, 165803 (2025). 10.1016/j.cej.2025.165803

[15] M. Ding, S. Chen, T. Xue, M. Xie, J. Weng et al., A novel carbon-free 3D porous honeycomb-like MoS_2_/ReS_2_ heterostructure with S vacancies as anodes for sodium-ion batteries. Green Chem. **27**(36), 11155–11166 (2025). 10.1039/d5gc02014j

[16] B. Ye, X. Cai, R. Zhao, Construction of hierarchical C/MoS_2_ nanobelts wrapped by N-doped carbon toward high-performance lithium/sodium storage. J. Colloid Interface Sci. **700**(Pt 2), 138458 (2025). 10.1016/j.jcis.2025.138458 40706360 10.1016/j.jcis.2025.138458

[17] B. Li, W. Cao, S. Wang, Z. Cao, Y. Shi et al., N, S-doped porous carbon nanobelts embedded with MoS_2_ nanosheets as a self-standing host for dendrite-free Li metal anodes. Adv. Sci. **9**(32), 2204232 (2022). 10.1002/advs.202204232 10.1002/advs.202204232 PMC9661841 36161278

[18] D. Sun, S. Lin, S. Kuai, T. Zhang, L. Liu et al., Interfacial Mo–N bonding enhancement of N-doped carbon nanosheets-stabilized ultrafine MoS_2_ enable ultrafast and durable sodium ion half/full batteries. Chem. Eng. J. **501**, 157786 (2024). 10.1016/j.cej.2024.157786

[19] G. Liu, T. Yan, Y. Zhang, P. Zeng, B. Wang et al., Enhanced basal-plane catalytic activity of MoS_2_ by constructing an electron bridge for high-performance lithium–sulfur batteries. Nano Lett. **24**(50), 15973–15980 (2024). 10.1021/acs.nanolett.4c04139 39651781 10.1021/acs.nanolett.4c04139

[20] J. Wang, T. Liu, B. Chen, Z. Qi, H. Xie et al., Engineering the catalytic superlattices for highly reversible sodium-ion storage with a high compositional conversion degree. Angew. Chem. Int. Ed. **64**(16), e202425063 (2025). 10.1002/anie.202425063 10.1002/anie.202425063 39924454

[21] Y. Xia, T. Yang, Z. Wang, T. Mao, Z. Hong et al., Van der Waals forces between S and P ions at the CoP–C@MoS_2_/C heterointerface with enhanced lithium/sodium storage. Adv. Funct. Mater. **33**(35), 2302830 (2023). 10.1002/adfm.202302830

[22] L. Ma, B. Zhao, X. Wang, J. Yang, X. Zhang et al., MoS_2_ nanosheets vertically grown on carbonized corn stalks as lithium-ion battery anode. ACS Appl. Mater. Interfaces **10**(26), 22067–22073 (2018). 10.1021/acsami.8b04170 29901387 10.1021/acsami.8b04170

[23] T. Wang, M. Li, L. Qi, P. Jie, W. Yang et al., Multilevel heterostructure of MoS_2_/GDYO for lithium-ion batteries. Adv. Funct. Mater. **33**(50), 2308470 (2023). 10.1002/adfm.202308470

[24] T. Ge, Y. Wang, J. Xu, Investigation of the lithium storage enhancement mechanism in IL-MoS_2_@C/rGO hierarchical material induced by [BMIM] HSO_4_ self-assembly. Electrochim. Acta **521**, 145940 (2025). 10.1016/j.electacta.2025.145940

[25] S. Huang, Y. Cao, F. Yao, D. Zhang, J. Yang et al., Interface density engineering on heterogeneous molybdenum dichalcogenides enabling highly efficient hydrogen evolution catalysis and sodium ion storage. Small **19**(26), e2207919 (2023). 10.1002/smll.202207919 36938911 10.1002/smll.202207919

[26] S. Ali, M. Sufyan Javed, K. Umer, J. Wang, Y. Fu et al., MoS_2_@Ti_3_C_2_T_*x*_ heterostructure: a new negative electrode material for Li-Ion hybrid supercapacitors. Chem. Eng. J. **498**, 155330 (2024). 10.1016/j.cej.2024.155330

[27] S. Huang, Y. Cao, C. Liang, M. Li, H. Yao et al., Oxygen doping-triggered electron redistribution in cobalt-rich sulfide for efficient electrocatalytic water splitting. J. Colloid Interface Sci. **690**, 137382 (2025). 10.1016/j.jcis.2025.137382 40121841 10.1016/j.jcis.2025.137382

[28] Y. Cao, Y. Meng, S. Huang, S. He, X. Li et al., Nitrogen-, oxygen- and sulfur-doped carbon-encapsulated Ni_3_S_2_ and NiS core–shell architectures: bifunctional electrocatalysts for hydrogen evolution and oxygen reduction reactions. ACS Sustain. Chem. Eng. **6**(11), 15582–15590 (2018). 10.1021/acssuschemeng.8b04029

[29] J. Li, L. Han, X. Zhang, H. Sun, X. Liu et al., Multi-role TiO_2_ layer coated carbon@few-layered MoS_2_ nanotubes for durable lithium storage. Chem. Eng. J. **406**, 126873 (2021). 10.1016/j.cej.2020.126873

[30] J. Zhong, S. Huang, Y. Cao, K. Pei, D. Zhang et al., Inner-wrinkled porous carbons via soft-hard coupling assembly strategy for ultrahigh-capacity lithium-ion batteries. Adv. Funct. Mater. **36**(5), e14143 (2026). 10.1002/adfm.202514143

[31] K. Pei, S. Huang, Y. Cao, J. Zhong, M. Li et al., Spongy silicon-doped MoS_2_*via* long-chain molecule induction and mesopore confinement for ultra-stable lithium-ion storage. Adv. Energy Mater. **15**(23), 2500119 (2025). 10.1002/aenm.202500119

[32] B. Lan, X. Zhang, Y. Wang, C. Wei, G. Wen, Constructing highly stable lithium storage materials by improving the bond strength of MoS_2_ to graphene via chitosan. Carbon **192**, 384–394 (2022). 10.1016/j.carbon.2022.03.014

[33] C. Sun, M. Liu, L. Wang, L. Xie, W. Zhao et al., Revisiting lithium-storage mechanisms of molybdenum disulfide. Chin. Chem. Lett. **33**(4), 1779–1797 (2022). 10.1016/j.cclet.2021.08.052

[34] L. Yu, X. He, L. Tang, X. Wang, W. Cai et al., Understanding mechanisms of fast sodium storage kinetics for MXene/MoS_2_@C in ether electrolytes. J. Power. Sources **641**, 236852 (2025). 10.1016/j.jpowsour.2025.236852

[35] W. Liu, D. Fan, W. Wang, S. Yang, Y. Lu et al., One-pot hydrothermal synthesis and electrochemical performance of subspheroidal core–shell structure MoS_2_/C composite as anode material for lithium-ion batteries. Energies **17**(7), 1678 (2024). 10.3390/en17071678

[36] J. Ren, H. Guo, Z. Wang, G. Ling, J. Han et al., Engineering of single atomic Fe–N_4_ sites on hollow carbon cages to achieve highly reversible MoS_2_ anodes for Li-ion batteries. J. Colloid Interface Sci. **664**, 45–52 (2024). 10.1016/j.jcis.2024.03.023 38458054 10.1016/j.jcis.2024.03.023

[37] D. Li, G. Lin, Z. Huang, X. Tang, Z. Wu et al., Effect of different shell structure on lithium storage properties of MoS_2_ anode. J. Electroanal. Chem. **905**, 115972 (2022). 10.1016/j.jelechem.2021.115972

[38] Z. Liu, H. Li, Z. Gao, L. Bi, M. Qi et al., Smart construction of MoS₂ on carbon cloth flexible electrodes as high-performance anode for lithium-ion and sodium-ion batteries. ChemistrySelect **10**(12), e202405597 (2025). 10.1002/slct.202405597

[39] J. Pan, Z. Liu, B. Zhang, M. Qi, Y. Feng, Embedment of molybdenum disulfide in electrospun fibers as an integrated cathode for lithium-ion batteries. Coatings **14**(11), 1465 (2024). 10.3390/coatings14111465

[40] S. Tao, R. Momen, Z. Luo, Y. Zhu, X. Xiao et al., Trapping lithium selenides with evolving heterogeneous interfaces for high-power lithium-ion capacitors. Small **19**(15), 2207975 (2023). 10.1002/smll.202207975 10.1002/smll.202207975 36631278

[41] L. Wei, S. Geng, H. Liu, L. Deng, Y. Mao et al., Crystallographic engineering enables fast low-temperature ion transport of TiNb_2_O_7_ for cold-region lithium-ion batteries. Nano-Micro Lett. **18**(1), 91 (2026). 10.1007/s40820-025-01949-0 10.1007/s40820-025-01949-0 PMC12756213 41476275

[42] Y. Lu, C.-Z. Zhao, J.-Q. Huang, Q. Zhang, The timescale identification decoupling complicated kinetic processes in lithium batteries. Joule **6**(6), 1172–1198 (2022). 10.1016/j.joule.2022.05.005

[43] J. Wang, D. Zhang, Q. Wang, Q. Sun, H. Sun et al., Carbon mediated multifunctional Sb_2_Se_3_–WSe_2_ heterostructure nanofiber facilitates rapid and stable Na^+^ transport. Adv. Funct. Mater. **34**(28), 2400261 (2024). 10.1002/adfm.202400261

[44] X. Wang, Y. Liu, Z. Wei, J. Hong, H. Liang et al., MXene-boosted imine cathodes with extended conjugated structure for aqueous zinc-ion batteries. Adv. Mater. **34**(50), 2206812 (2022). 10.1002/adma.202206812 10.1002/adma.202206812 36269022

[45] J. Jiang, Y. Zhang, Y. An, L. Wu, Q. Zhu et al., Engineering ultrathin MoS_2_ nanosheets anchored on N-doped carbon microspheres with pseudocapacitive properties for high-performance lithium-ion capacitors. Small Methods **3**(7), 1900081 (2019). 10.1002/smtd.201900081

[46] J. Chao, L. Yang, H. Zhang, J. Liu, R. Hu et al., Engineering layer structure of MoS_2_/polyaniline/graphene nanocomposites to achieve fast and reversible lithium storage for high energy density aqueous lithium-ion capacitors. J. Power. Sources **450**, 227680 (2020). 10.1016/j.jpowsour.2019.227680

[47] D.T. Pham, J.P. Baboo, J. Song, S. Kim, J. Jo et al., Facile synthesis of pyrite (FeS_2_/C) nanoparticles as an electrode material for non-aqueous hybrid electrochemical capacitors. Nanoscale **10**(13), 5938–5949 (2018). 10.1039/c7nr06352k 29542744 10.1039/c7nr06352k

[48] Z.-C. Lu, J. Liu, L.-B. Kong, Construction of MoSe_2_ nanoparticles anchored on layered microporous carbon heterostructure anode for high-performance and low-cost lithium-ion capacitors. Solid State Ion. **374**, 115815 (2022). 10.1016/j.ssi.2021.115815

[49] H.-J. Zhang, Y.-K. Wang, L.-B. Kong, A facile strategy for the synthesis of three-dimensional heterostructure self-assembled MoSe_2_ nanosheets and their application as an anode for high-energy lithium-ion hybrid capacitors. Nanoscale **11**(15), 7263–7276 (2019). 10.1039/c9nr00164f 30932121 10.1039/c9nr00164f

[50] A. Chaturvedi, P. Hu, V. Aravindan, C. Kloc, S. Madhavi, Unveiling two-dimensional TiS_2_ as an insertion host for the construction of high energy Li-ion capacitors. J. Mater. Chem. A **5**(19), 9177–9181 (2017). 10.1039/c7ta01594a

