# Supplementary material for: Surfactant-Derived Nitrogen-Bridged MoS2/C Heterostructures for Robust Lithium-Ion Storage
Source: Nanomicro Lett. 2026 Aug 23;19:38. doi: 10.1007/s40820-026-02324-3 (PMC13499779; doi:10.1007/s40820-026-02324-3)
Supplement: Supplementary file 1 [file 40820_2026_2324_MOESM1_ESM.docx]

Supporting Information for

**Surfactant-Derived Nitrogen-Bridged MoS_2_/C Heterostructures for Robust Lithium-Ion Storage**

Senchuan Huang^1, #,^ *, Kewei Pei^1, #^, Yunyi Chen^1^, Yangfei Cao^2,^ *, Jingwen Shangguan^1^, Shiman He^3^, Junxia Meng^4^, and Shanqing Zhang^1, 5,^ *

^1^ School of Chemical Engineering and Light Industry, Guangdong University of Technology, Guangzhou 510006, P. R. China

^2^ School of Materials and Energy, Guangdong University of Technology, Guangzhou 510006, P. R. China

^3^ School of Materials Science and Engineering, Guangdong Provincial Key Laboratory of Advanced Energy Storage Materials, South China University of Technology, Guangzhou 510641, P. R. China

^4^ School of Physics and Electronics, Gannan Normal University, Ganzhou 341000, P. R. China

^5^ School of Environment and Science, Griﬃth University, Gold Coast, QLD 4222, Australia

^#^ Senchuan Huang and Kewei Pei contribute to the work equally.

* Corresponding authors. E-mail: [huangsch@gdut.edu.cn](mailto:huangsch@gdut.edu.cn) (Senchuan Huang); [caoyf@gdut.edu.cn](mailto:caoyf@gdut.edu.cn) (Yangfei Cao); [s.zhang@griffith.edu.au](mailto:s.zhang@griffith.edu.au) (Shanqing Zhang)

**Supplementary Figures and Tables**

**Fig. S1** FESEM image of mSiO_2_@CTAB

**Fig. S2** **a** Nitrogen adsorption–desorption isotherms and **b** corresponding pore size distribution curve of mSiO_2_@CTAB

**Fig. S3** FESEM images of SiO_2_@MoS_2_-N-C composites at different magnifications: **a, b** SiO_2_@MoS_2_-N-C_600_, **c, d** SiO_2_@MoS_2_-N-C_700_, and **e, f** SiO_2_@MoS_2_-N-C_800_

**Fig. S4** Digital photographs of mSiO_2_@CTAB before and after carbonization

**Fig. S5** TG curve of CTAB measured under Ar

**Fig. S6** TG curve of thiourea measured under Ar

**Fig. S7** FESEM images of the MoS_2_-N-C heterostructures synthesized at different temperatures: **a** MoS_2_-N-C_600_ and **b** MoS_2_-N-C_800_

**Fig. S8** **a** TEM image and **b** SAED pattern of MoS_2_-N-C_700_

**Fig. S9** FESEM images of commercial MoS_2_ at different magnifications

**Fig. S10** EDS spectrum of MoS_2_-N-C_700_, showing the existence of N-dopants

**Fig. S11** **a** TEM and **b** HRTEM images of commercial MoS_2_

**Fig. S12** XRD pattern of commercial MoS_2_

**Fig. S13** Full Raman spectra of MoS_2_-N-C_600_, MoS_2_-N-C_700_, and MoS_2_-N-C_800_

**Fig. S14** Pore size distribution curves of MoS_2_-N-C_600_, MoS_2_-N-C_700_, and MoS_2_-N-C_800_

**Fig. S15** XPS survey spectra of MoS_2_-N-C_600_, MoS_2_-N-C_700_, and MoS_2_-N-C_800_

**Fig. S16** **a** Normalized XANES spectrum and **b** Fourier transformed *k*^2^-weighted EXAFS spectrum of Mo *K*-edge for MoS_2_-N-C_700_

**Fig. S17** Structure characterization of Pristine MoS_2_ control sample. **a, b** FESEM images at different magnifications, **c** XRD pattern, and **d** TG curves

**Fig. S18** **a** N_2_ adsorption-desorption isotherms and **b-d** Mo 3d, Mo 3p and S 2p spectra of Pristine MoS_2_ control sample, respectively

**Fig. S19** EPR spectra of MoS_2_-N-C_600_, MoS_2_-N-C_700_, and MoS_2_-N-C_800_

**Fig. S20** EPR tests of MoS_2_-N-C_700_ electrodes after different cycles at 0.5 A g^-1^

**Fig. S21** **a** N_2_ absorption/desorption isotherms and **b** relative specific area of MoS_2_-N-C_700_ electrodes before cycling and after different cycles at 0.5 A g^-1^

**Fig. S22** **a** CV cures and **b** corresponding redox peak currents of MoS_2_-N-C_700_ electrodes after different cycles

**Fig. S23** Deconvoluted XPS spectra of MoS_2_-N-C_700_ electrodes after different cycles at 0.5 A g^-1^: **a** Mo 3d, and **b** N 1s + Mo 3p

**Fig. S24** Cycling stability of MoS_2_-N-C_600_ at 0.5 A g^-1^

**Fig. S25** Cycling stability of MoS_2_-N-C_800_ at 0.5 A g^-1^

**Fig. S26 a** N_2_ adsorption-desorption isotherms and **b** corresponding pore size distribution curve of MoS_2_/NC control sample

**Fig. S27** SEM images of MoS_2_/NC control sample at different magnifications

**Fig. S28** Cycling stability of MoS_2_-N-C_700_ at 2 A g^-1^

**Fig. S29** d*Q*/d*V* curves at various cycles of **a** MoS_2_-N-C_600_ and **b** MoS_2_-N-C_800_

**Fig. S30** d*Q*/d*V* curves of MoS_2_/NC at various cycles

**Fig. S31** First three CV curves at 0.1 mV s^-1^ of **a** MoS_2_-N-C_600_ and **b** MoS_2_-N-C_800_

**Fig. S32** First three CV curves at 0.1 mV s^-1^ of commercial MoS_2_

**Fig. S33** First three CV curves at 0.1 mV s^-1^ of MoS_2_/NC

**Fig. S34** First three GCD curves of **a** MoS_2_-N-C_600_ and **b** MoS_2_-N-C_800_ obtained at 0.1 A g^-1^

**Fig. S35** First three GCD curves of **a** commercial MoS_2_ and **b** MoS_2_/NC obtained at 0.1 A g^-1^

**Fig. S36 a** Rate performance and **b** cycling stability at 0.5 A g^-1^ of MoS_2_-N-C_700_ at a high mass loading of ~2.9 mg cm^-2^

**Fig. S37** Rate performance of MoS_2_-N-C_700_, commercial MoS_2_ and MoS_2_/NC electrodes

**Fig. S38** Deconvoluted N 1s XPS spectra for the MoS_2_-N-C_700_ electrode after discharging to 0.01 V at 0.1 A g^-1^

**Fig. S39** Nyquist plots of MoS_2_-N-C_600_, MoS_2_-N-C_700_ and MoS_2_-N-C_800_

**Fig. S40** Linear relationship between *Z*′ and 𝜔^-1/2^ of MoS_2_-N-C_600_, MoS_2_-N-C_700_, and MoS_2_-N-C_800_

**Fig. S41** Nyquist plot of MoS_2_/NC control sample

**Fig. S42** **a** CV curves at scan rates from 0.2 to 1.0 mV s^-1^, **b** linear fitting of the logarithmic relationship between peak current log(*i*) and scan rate log(*v*), **c** pseudocapacitive contribution at different scan rates, and **d** pseudocapacitive contribution at 1.0 mV s^-1^ of MoS_2_-N-C_600_

**Fig. S43** **a** CV curves at scan rates from 0.2 to 1.0 mV s^-1^, **b** linear fitting of the logarithmic relationship between peak current log(*i*) and scan rate log(*v*), **c** pseudocapacitive contribution at different scan rates, and **d** pseudocapacitive contribution at 1.0 mV s^-1^ of MoS_2_-N-C_800_

**Fig. S44** Pseudocapacitive contribution of MoS_2_-N-C_700_ at different scan rates

**Fig. S45** **a** CV curves at scan rates from 0.2 to 1.0 mV s^-1^, **b** linear fitting of the logarithmic relationship between peak current log(*i*) and scan rate log(*v*), **c** pseudocapacitive contribution at different scan rates, and **d** pseudocapacitive contribution at 1.0 mV s^-1^ of MoS_2_/NC

**Fig. S46** Equivalent circuits for EIS spectra fitting

**Fig. S47** The trends of **a** *R*_ct_ and **b** *D*_Li+_ values obtained from *in-situ* EIS spectra during discharge and charge for MoS_2_-N-C_700_

**Fig. S48** *In-situ* EIS spectra of MoS_2_-N-C_600_ during the 6^th^ cycle: **a** discharge process and **b** charge process

**Fig. S49** *In-situ* EIS spectra of MoS_2_-N-C_800_ during the 6^th^ cycle: **a** discharge process and **b** charge process

**Fig. S50** DRT plots of MoS_2_-N-C_600_ based on the EIS spectra: **a** discharge process and **b** charge process

**Fig. S51** DRT plots of MoS_2_-N-C_700_ based on the EIS spectra: **a** discharge process and **b** charge process

**Fig. S52** DRT plots of MoS_2_-N-C_800_ based on the EIS spectra: **a** discharge process and **b** charge process

**Fig. S53** XRD pattern of the as-synthesized WS_2_-N-C_700_ heterostructure

**Fig. S54** FESEM image of the as-synthesized WS_2_-N-C_700_ heterostructure

**Fig. S55** Electrochemical performance of the as-synthesized WS_2_-N-C_700_ heterostructure for LIBs. **a** CV curves, **b** rate capability, **c** GCD profiles, and **d** cycling stability at 0.5A g^-1^

**Table S1** Quantitative analysis of different chemical states of S species obtained from the deconvoluted XPS spectra

| **Samples** | **Chemical states** | | **Peak position (eV)** | **Relative percentage (%)** |
| --- | --- | --- | --- | --- |
| **MoS_2_-N-C_600_** | Sulphate | 2p 1/2 | 169.7 | 16.8 |
|  |  | 2p 3/2 | 168.6 |  |
|  | C-S | 2p 1/2 | 165.2 | 10.2 |
|  |  | 2p 3/2 | 164.1 |  |
|  | Mo-S | 2p 1/2 | 163.5 | 73.0 |
|  |  | 2p 3/2 | 162.4 |  |
| **MoS_2_-N-C_700_** | Sulphate | 2p 1/2 | 169.8 | 19.8 |
|  |  | 2p 3/2 | 168.7 |  |
|  | C-S | 2p 1/2 | 165.2 | 6.0 |
|  |  | 2p 3/2 | 164.1 |  |
|  | Mo-S | 2p 1/2 | 163.5 | 74.2 |
|  |  | 2p 3/2 | 162.4 |  |
| **MoS_2_-N-C_800_** | Sulphate | 2p 1/2 | 169.9 | 12.4 |
|  |  | 2p 3/2 | 168.8 |  |
|  | C-S | 2p 1/2 | 165.2 | 2.6 |
|  |  | 2p 3/2 | 164.1 |  |
|  | Mo-S | 2p 1/2 | 163.6 | 85.0 |
|  |  | 2p 3/2 | 162.5 |  |

**Table S2** Relative percentages of nitrogen species in the MoS_2_-N-C heterostructures determined from the deconvoluted N 1s XPS spectra

| **Samples** | **Chemical states** | **Peak position (eV)** | **Relative percentage (%)** |
| --- | --- | --- | --- |
| **MoS_2_-N-C_600_** | Mo–N | 397.5 | 17.2 |
|  | Pyridinic N | 398.5 | 33.0 |
|  | Pyrrolic N | 399.9 | 30.4 |
|  | Graphitic N | 401.2 | 19.4 |
| **MoS_2_-N-C_700_** | Mo–N | 397.6 | 16.4 |
|  | Pyridinic N | 398.5 | 30.0 |
|  | Pyrrolic N | 399.8 | 30.4 |
|  | Graphitic N | 401.3 | 23.1 |
| **MoS_2_-N-C_800_** | Mo–N | 397.6 | 15.1 |
|  | Pyridinic N | 398.5 | 33.6 |
|  | Pyrrolic N | 399.8 | 26.1 |
|  | Graphitic N | 401.4 | 25.2 |

**Table S3** Charge transfer resistance (*R*_ct_) and Li^+^ diffusion coefficient (*D*_Li+_) values obtained from fitting the EIS spectra

|  | **MoS_2_-N-C_600_** | **MoS_2_-N-C_700_** | **MoS_2_-N-C_800_** |
| --- | --- | --- | --- |
| ***R*_ct_ (ohm)** | 106.7 | 77.1 | 83.1 |
| ***D*_Li+_ (10^-14^ cm^2^ s^-1^)** | 1.2 | 3.8 | 1.4 |

**Table S4** Cycling performance comparison of recent state-of-the-art MoS_2_/C anodes for LIBs

| **Anode** | **Mass loading (mg cm^-2^)** | **Current density (A g^-1^)** | **Cycle number** | **Capacity retention** | **Ref.** |
| --- | --- | --- | --- | --- | --- |
| MoS_2_-N-C_700_ | 1.1~1.3 | 0.5 | 800 | ~100% | **This work** |
| MoS_2_@C | ~2.0 | 1 | 100 | 21.7% | [S1] |
| NG@MoS_2_@LS-C | ~3.1 | 0.1 | 100 | 55.5% | [S2] |
| B-MoS_2_–GO | ~0.6 | 0.5 | 200 | 77.9% | [S3] |
| Ni-MoS_2_/rGO | ~2.0 | 0.1 | 100 | 79.0% | [S4] |
| Co_3_S_4_/1T-MoS_2_@NC | 1.0~1.1 | 1 | 250 | 75.7% | [S5] |
| 1T/2H hybrid phase MoS_2_-graphene | 2~3 | 0.18 | 100 | 90.0% | [S6] |
| MoS_2_/C-3 | 1.6~2.1 | 1 | 1300 | 41.4% | [S7] |
| 5% BN-G@MoS_2_-50@50 | 0.57~0.85 | 1 | 500 | 85.6% | [S8] |
| SnS_2_/MoS_2_/DGS | ~2.0 | 0.2 | 200 | 96.9% | [S9] |

**Table S5** Comparisons of electrochemical performance of carbon-based LISCs reported previously

| **System** | **Voltage window** (V) | **Current density (A g^-1^)** | **Energy density (Wh kg^-1^)** | **Cycling performance** | **Ref.** |
| --- | --- | --- | --- | --- | --- |
| MoS_2_-N-C_700_//AC | 0.5-4 | 0.1 | 175.0 | 1 A g^-1^@10000 cycles@78.2% | **This work** |
| MC800//HC | 2-4 | 0.25 | 152.2 | 2 A g^-1^@5000 cycles@80.0% | [S10] |
| Zn_80_Co_20_-PC//AC | 2-4 | 0.05 | 123.0 | 1 A g^-1^@5000 cycles@90.0% | [S11] |
| CAC//NPC | 0.01-4 | 1 | 151.8 | 2 A g^-1^@1600 cycles@87.0% | [S12] |
| NPC//AC | 2.0-4.2 | 0.1 | 112.0 | 1 A g-1@6000 cycles@72.0% | [S13] |
| CTK-1.0//CTK-1.0 | 0.1-4.2 | 0.1 | 137.6 | 2 A g^-1^@3000 cycles@75.0% | [S14] |
| NP-HPC//NP-SC | 1-3.8 | 0.1 | 121.0 | 1 A g^-1^@3000 cycles@80.2% | [S15] |
| PCNs-800//PCNs-800 | 1-4.2 | 0.1 | 112.0 | 1 A g^-1^@9000 cycles@63.0% | [S16] |
| NHCM-12//NHCM-A | 0.1-4 | 0.1 | 162.0 | 1 A g^-1^@5000 cycles@86.2% | [S17] |
| DGPC-1//AC | 2-4 | 0.1 | 103.4 | 5 A g^-1^@5000 cycles@70.2% | [S18] |

**Supplementary References**

[S1] D. Li, G. Lin, Z. Huang, X. Tang, Z. Wu et al., Effect of different shell structure on lithium storage properties of MoS_2_ anode. J. Electroanal. Chem. **905**, 115972 (2022). <https://doi.org/10.1016/j.jelechem.2021.115972>
[S2] J. Sun, Z. Xu, W. Wang, S. Sheng, S. Zhao et al., Preparation and electrochemical properties of mesoporous Carbon@ MoS_2_ anode materials for lithium-ion batteries. Int. J. Electrochem. Sci. **21**(3), 101294 (2026). <https://doi.org/10.1016/j.ijoes.2026.101294>
[S3] H. Lu, B. Wu, Y. Zhang, T. Dong, Q. Duan et al., A simple and scalable method for preparing natural molybdenite-based MoS_2_–graphene oxide composite anodes *via* ultrasonic exfoliation combined with a solvothermal process. J. Electron. Mater. **55**(3), 2969–2987 (2026). <https://doi.org/10.1007/s11664-026-12686-4>
[S4] S. Zhang, K. Qi, B. Du, G. Zhu, X. Liu et al., Reduced graphene oxide supported Ni-doped MoS_2_ nanosheets with enhanced lithium-ion storage performance. J. Electroanal. Chem. **997**, 119501 (2025). <https://doi.org/10.1016/j.jelechem.2025.119501>
[S5] S.-C. Dong, R. Zeng, H. Zhang, S.-T. Zhang, P.-P. Chen et al., Enhancing stable and rapid Li^+^/Na^+^ storage performance *via* interface engineering of confining Co3S₄/1T-MoS₂ heterostructure on nitrogen-doped carbon. Electrochim. Acta **542**, 147415 (2025). <https://doi.org/10.1016/j.electacta.2025.147415>
[S6] K.Y. Jang, Y.A. Lee, S. Lim, S. Park, K.-N. Jung et al., Enhancing lithium-ion battery kinetics and stability leveraging hybrid 1T/2H MoS_2_–graphene heterostructures. Chem. Eng. J. **520**, 165803 (2025). <https://doi.org/10.1016/j.cej.2025.165803>
[S7] L. Liu, W. Du, Q. Zhang, H. Jiang, Y. Zhang et al., Constructing hollow flower-like molybdenum disulfide nanospheres/carbon nanospheres as anode with enhanced diffusion kinetics for lithium storage. Adv. Compos. Hybrid Mater. **7**(6), 195 (2024). <https://doi.org/10.1007/s42114-024-01029-8>
[S8] B. Chandra Sekhar, A. Soliman, M. Arsalan, E.H. Alsharaeh, A 2D hybrid nanocomposite: a promising anode material for lithium-ion batteries at high temperature. Nanoscale Adv. **6**(22), 5612–5624 (2024). <https://doi.org/10.1039/d4na00424h>
[S9] W. Yu, B. Cui, J. Han, S. Zhu, X. Xu et al., *In situ* encapsulation of SnS(2)/MoS_2_ heterojunctions by amphiphilic graphene for high-energy and ultrastable lithium-ion anodes. Adv. Sci. **11**(36), 2405135 (2024). <https://doi.org/10.1002/advs.202405135>
[S10] H. Liu, X. Zhang, C. Li, S. Zhao, Y. An et al., Self-templating synthesis of mesoporous carbon cathode materials for high-performance lithium-ion capacitors. ChemSusChem **18**(3), e202401365 (2025). <https://doi.org/10.1002/cssc.202401365>
[S11] Q. Tang, G. Chu, Y. Xie, C. Yang, F. Tang et al., Zn/Co bimetallic metal-organic framework derived carbon anode with enchanted rate capability for lithium-ion capacitors. Mater. Today Commun. **41**, 110224 (2024). <https://doi.org/10.1016/j.mtcomm.2024.110224>
[S12] Y. Yang, H. Du, A. Wang, C. Lu, D. Sun et al., Excellent capacitive storage performance of N-doped porous carbon derived from the orientation-guidance coupled with *in-situ* activation methodology. J. Colloid Interface Sci. **673**, 657–668 (2024). <https://doi.org/10.1016/j.jcis.2024.05.237>
[S13] H. Jing, M. Chen, X. Zhang, Y. Yang, X. Zheng et al., CO_2_-derived nitrogen doped porous carbon as advanced anodes for lithium-ion capacitors. J. Alloys Compd. **983**, 173937 (2024). <https://doi.org/10.1016/j.jallcom.2024.173937>
[S14] J. Jiang, Q. Shen, Z. Chen, S. Wang, Nitrogen-doped porous carbon derived from coal for high-performance dual-carbon lithium-ion capacitors. Nanomaterials **13**(18), 2525 (2023). <https://doi.org/10.3390/nano13182525>
[S15] C. Cao, H. Hu, J. Duan, G. Zeng, X. Cai et al., Phytic acid mediated N, P Co-doped hierarchical porous carbon with boosting capacitive storage for dual carbon lithium-ion capacitor. Electrochim. Acta **470**, 143310 (2023). <https://doi.org/10.1016/j.electacta.2023.143310>
[S16] T. Qian, Y. Huang, M. Zhang, Z. Xia, H. Liu et al., Non-corrosive and low-cost synthesis of hierarchically porous carbon frameworks for high-performance lithium-ion capacitors. Carbon **173**, 646–654 (2021). <https://doi.org/10.1016/j.carbon.2020.11.051>
[S17] J. Jiang, J. Yuan, P. Nie, Q. Zhu, C. Chen et al., Hierarchical N-doped hollow carbon microspheres as advanced materials for high-performance lithium-ion capacitors. J. Mater. Chem. A **8**(7), 3956–3966 (2020). <https://doi.org/10.1039/c9ta08676e>
[S18] G. Zhang, K. Sun, Y. Liu, D. Wu, H. Zhu et al., Double reaction initiated self-assembly process fabricated hard carbon with high power capability for lithium ion capacitor anodes. Appl. Surf. Sci. **609**, 155083 (2023). <https://doi.org/10.1016/j.apsusc.2022.155083>
